# Supplementary material for: Impact and characterization of serial structural variations across humans and great apes
Source: Nat Commun. 2024 Sep 13;15:8007. doi: 10.1038/s41467-024-52027-9 (PMC11393467; doi:10.1038/s41467-024-52027-9)
Supplement: Supplementary file 1 — Supplementary Information [file 41467_2024_52027_MOESM1_ESM.pdf]

Impact and characterization of serial structural variations across humans and great apes

Supplementary Figures

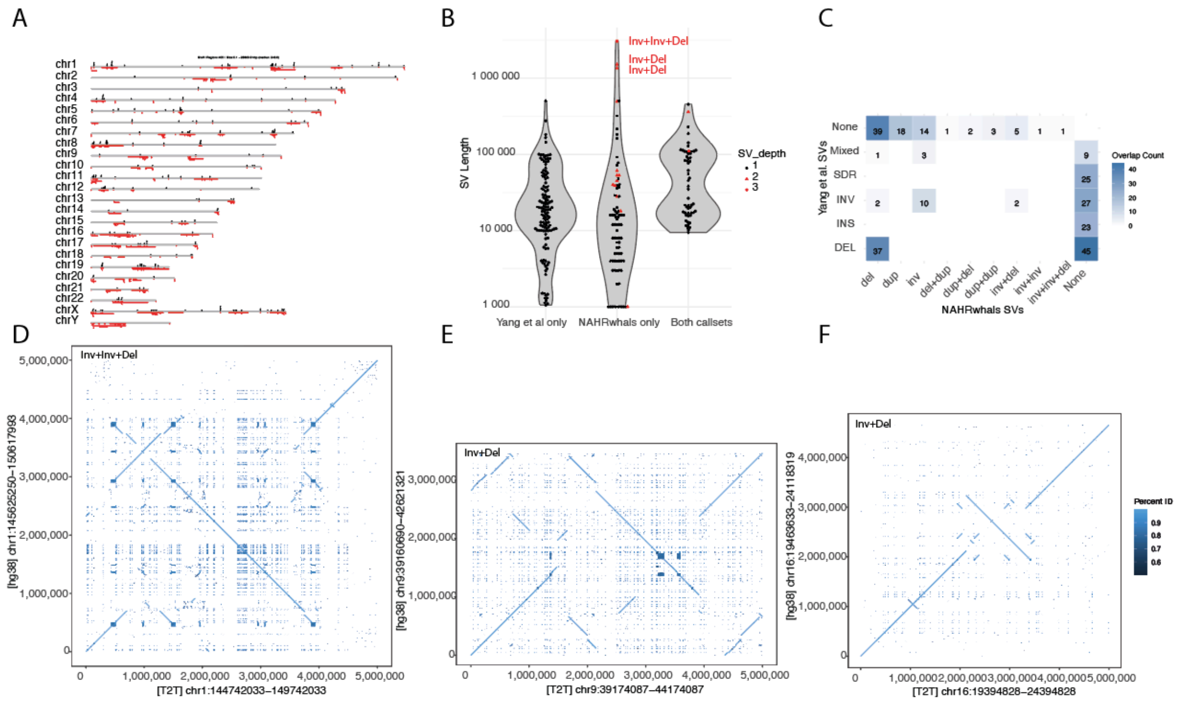

Figure S1. **Overview over a whole-genome run of the T2T vs hg38 reference genomes as a benchmark. A** NAHRwhals whole-genome-mode automatically selects windows of interest (red) based on an initial whole genome alignment. **B** A comparison of SV callsets between NAHRwhals and a gold-standard based on threees SV callers in [2]. Calls are considered common based on 25% reciprocal overlap. **C** Confusion matrix of the 55 overlapping calls. **D,E,F:** Dotplot views of the three largest SVs, which are all sSVs and exclusive to the NAHRwhals callset.

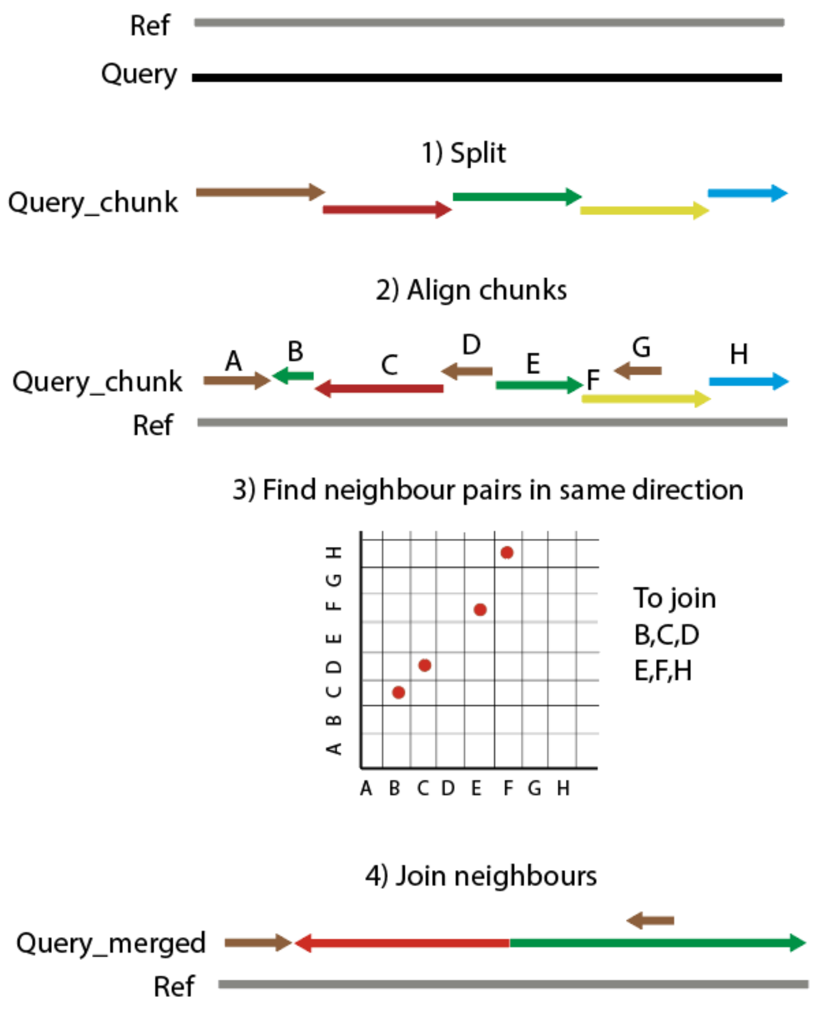

Figure S2. Schematic view of the custom alignment pipeline. (1) Before alignment, the Query sequence is split up into equality sized sequence 'chunks' of predefined length (100bp for sequences below 10 kbp, 1 kbp for sequences below 100 kbp, 10 kbp for sequences below 5 Mbp and 20 kbp above). **(2)** The chunks are aligned to the reference individually, using minimap2 aligner (version 2.18-r1035) [1] (methods). **(3)** Start- and end positions of all-vs-all alignments are then compared to identify pairs where the end of one coincides with the start of another alignment (methods). **(4)** all alignment pairs are then iteratively concatenated to create the final pairwise alignment.

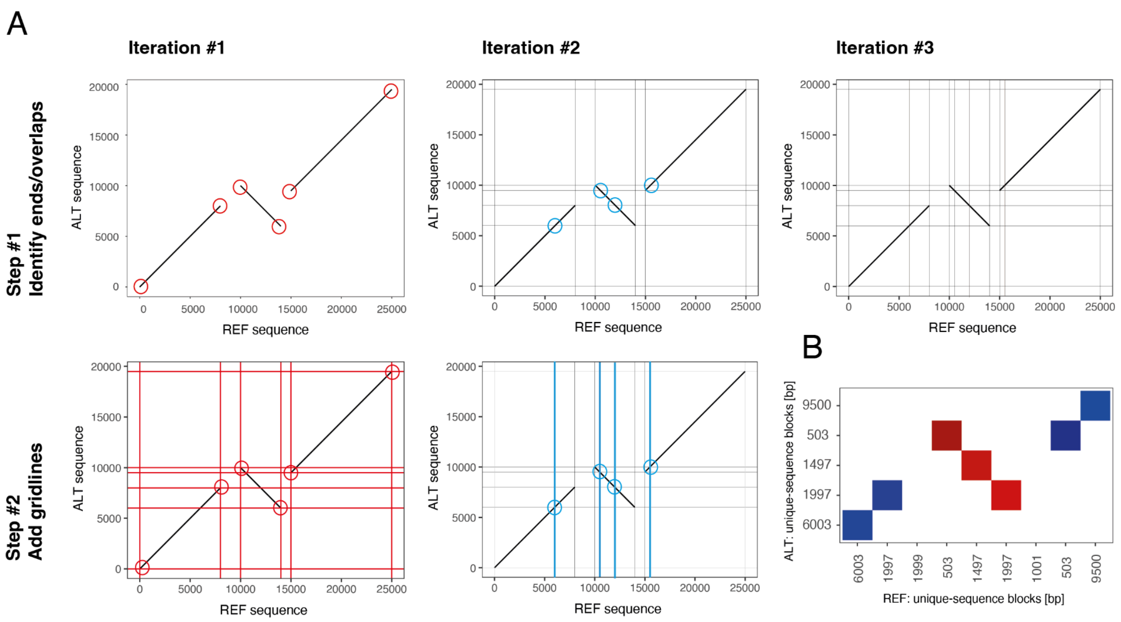

Figure S3. Visual representation of the iterative dotplot segmentation algorithm. A Starting from a pairwise alignment, initially all start-and endpoints are identified, and the x- and y-values are noted as the first set of 'gridlines' separating unique alignments. In each subsequent step, novel overlaps between existing gridlines and the pairwise alignments are identified, and subsequently new gridlines are inserted in x and y direction at the intersections. Once the grid has converged, each field is, by design, traversed by zero or one alignment vectors diagonally, intersecting with exactly two opposite corners. Grids which do not converge after 10 iterations are rejected and the dotplot pre-processing is repeated with another parameter set until a converging representation is found (Methods). **B** Using the determined grid as a reference, we derive a "condensed dotplot" where each field of the new dotplot represents a sector of the grid, the value represents the length of the traversing alignment, and the sign of the value corresponds to the direction of the alignment (blue: positive values: direct orientation; red: negative values: inverse orientation).

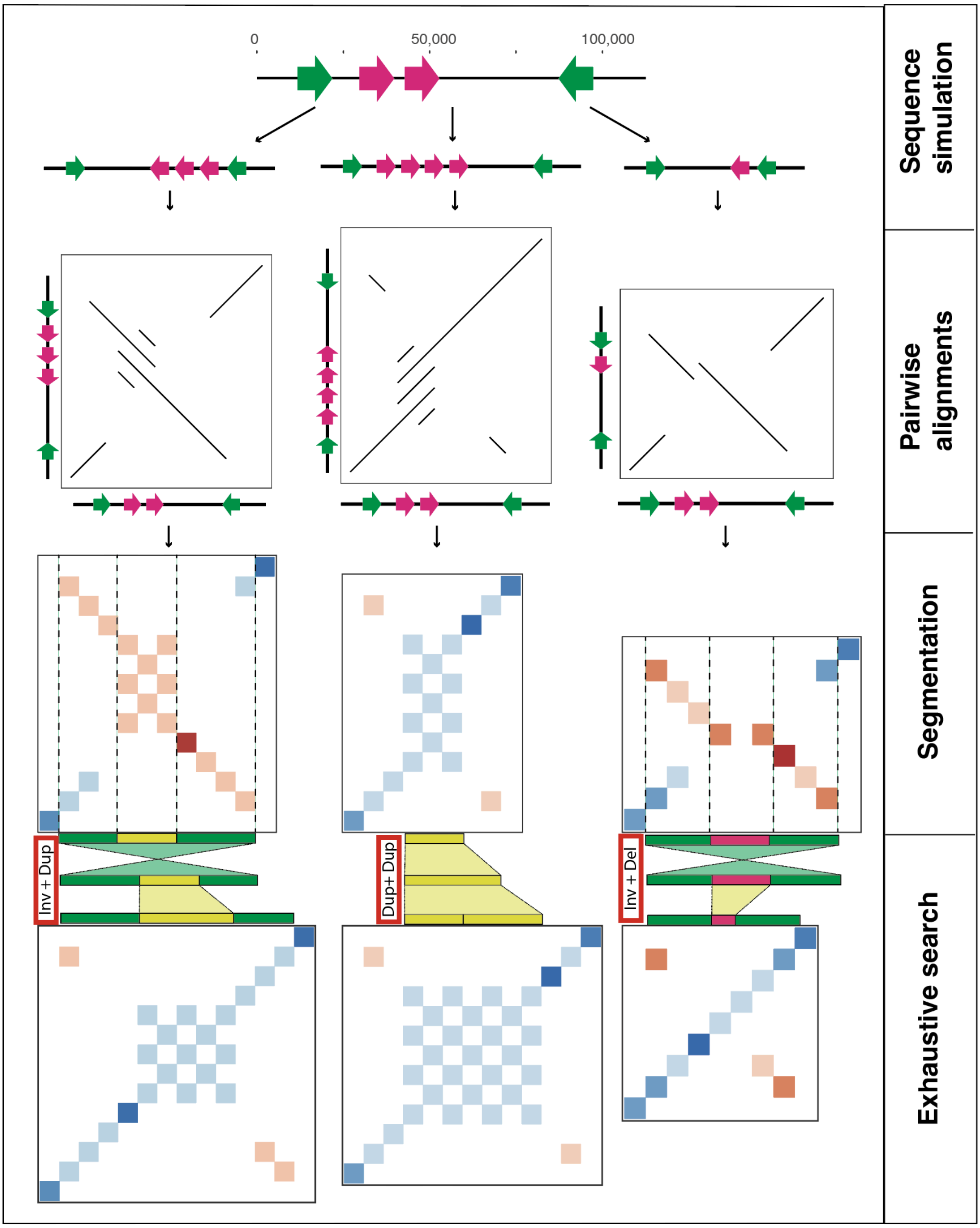

Figure S4. Workflow of simulation experiments. For each iteration in the simulation experiments, an artificial genomic region was created with two pairs of SDs, length and similarity of SDs as pre-defined parameters, and the position and orientation of SDs was randomly chosen each time. Simulated sequences were then subjected to in-silico mutation, applying all possible NAHR-chains of depth 1 and 2, resulting in three to >10 mutated ‘alternative’ alleles derived from one founder sequence. Each alternative sequence was tested for mutations using the NAHRwhal algorithm consisting of pairwise alignments, dotplot segmentation and exhaustive search. 50 genomic founder sequences were created for each combination of three SD similarities (90%, 95%, 99%) and four

different SD lengths (100 bp, 500 bp, 1.000 bp, 10.000 bp). The resulting SV calls were finally compared to the in-silico mutation ground truth.

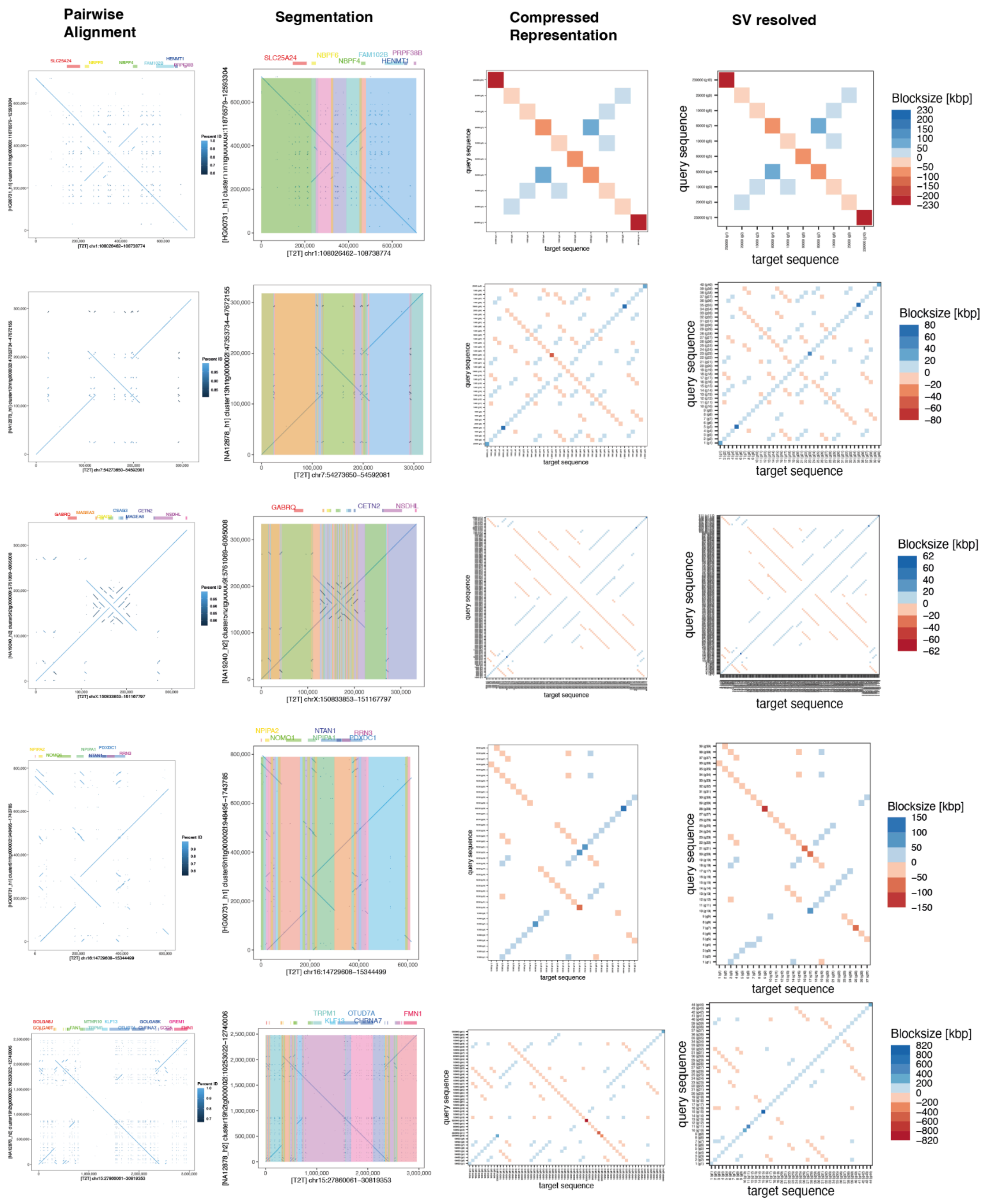

Figure S5. Unittest loci 1-5/10. Pairwise alignments (left), segmentations (plotted on x-axis only for clarity), condensed dotplots and mutation-resolved condensed dotplots for five example loci representing loci of different SD- and SV complexity. The segmentation algorithm scales window size dynamically depending on the complexity of a locus, leading to a fine representation of complex, repetitive regions in the compressed representation.

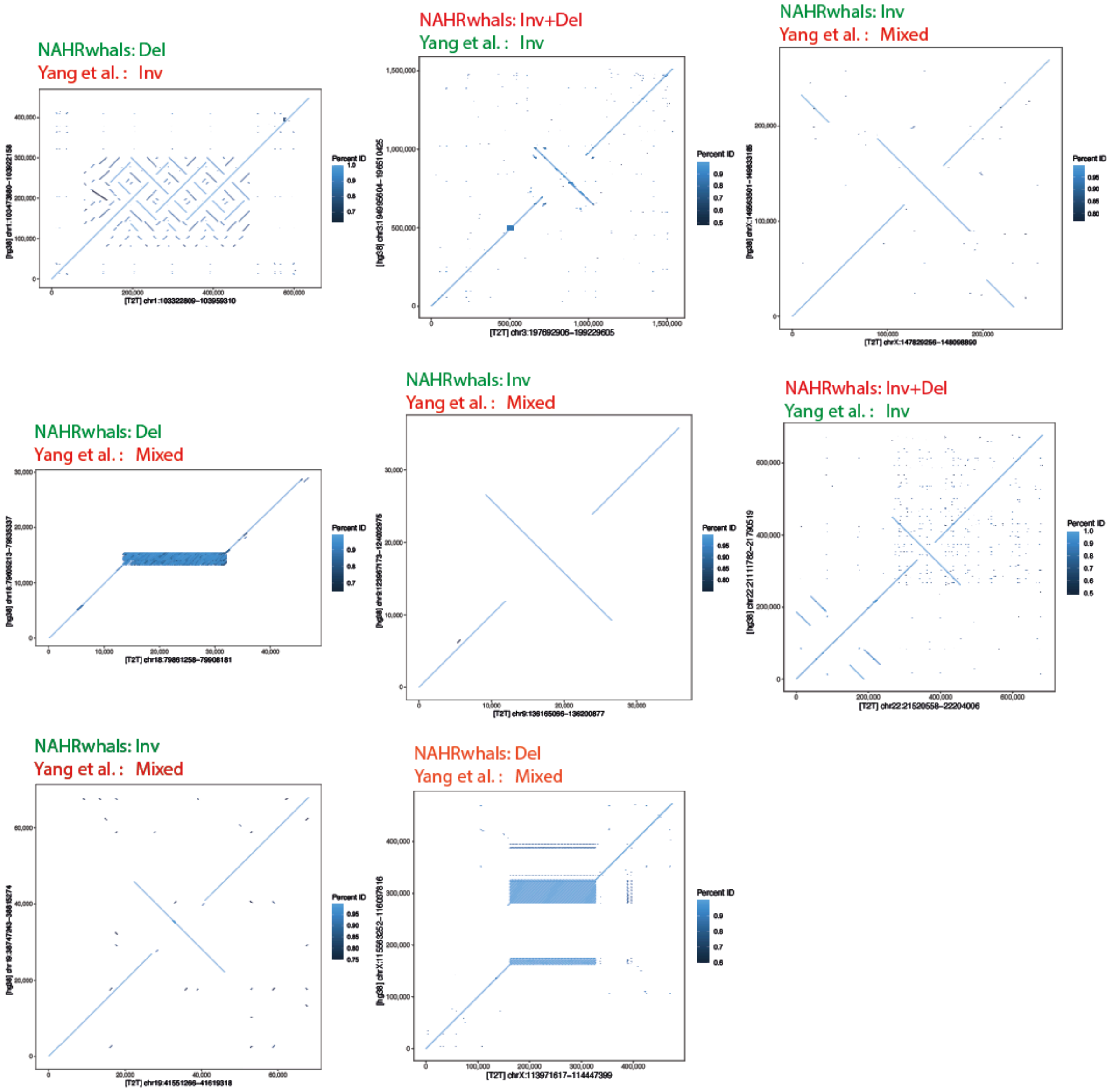

Fig S6. A view of the 8 SVs which were called both by NAHRwhals and Yang et al., but with different genotypes. Genotype assignments visually judged to be correct are highlighted in green (Nahrwhals: 5, Yang et al.: 2, undecided: 1).

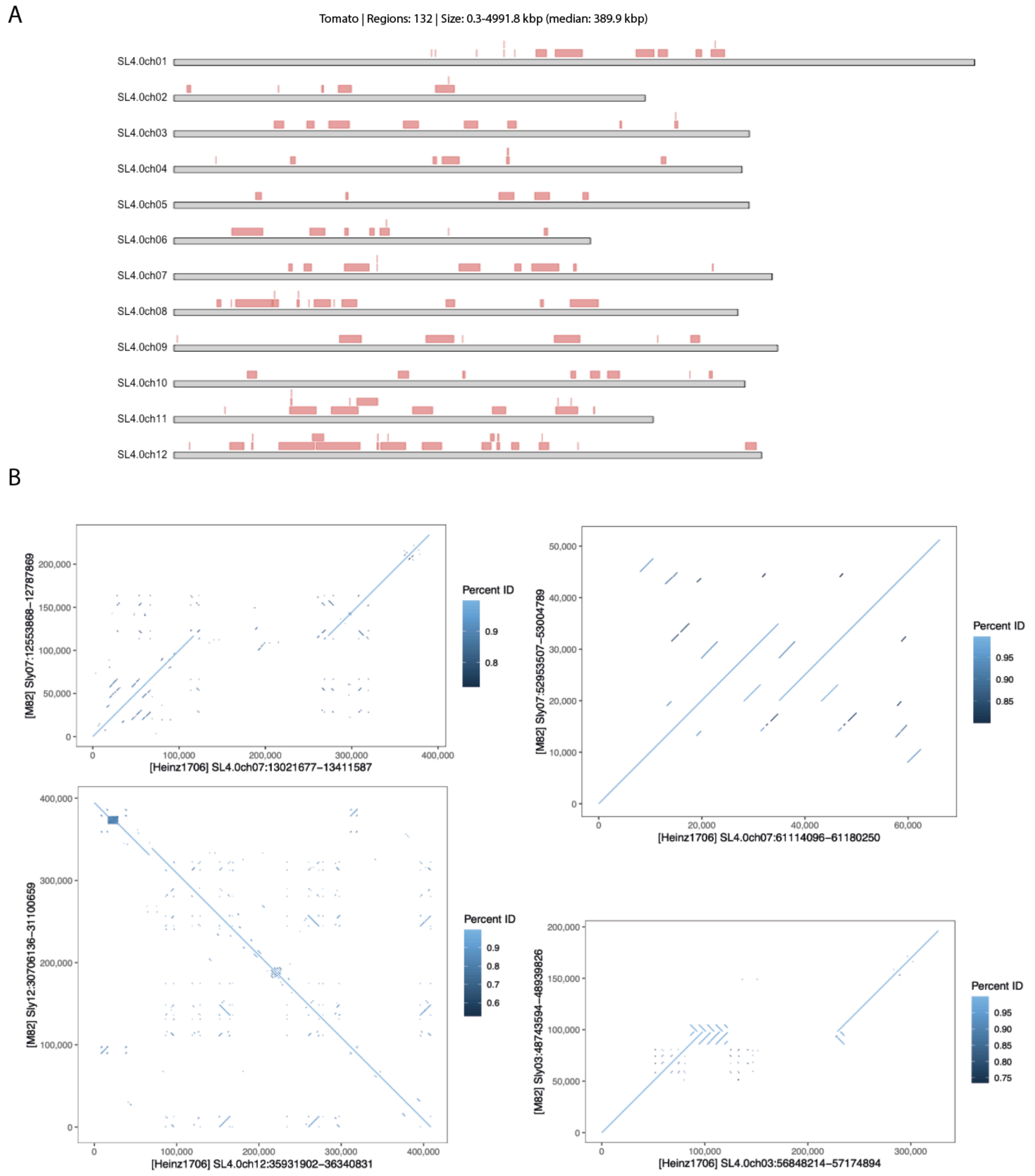

Figure S7. Results of running NAHRwhals in whole-genome mode on the Heinz1706 vs M82 Tomato plant assemblies. A Based on an initial whole-genome alignment, NAHRwhals selected 132 regions between 0.3 and 4992 kbp for NAHR-identification. **B** Dotplot view of the four identified NAHR-consistent SVs.

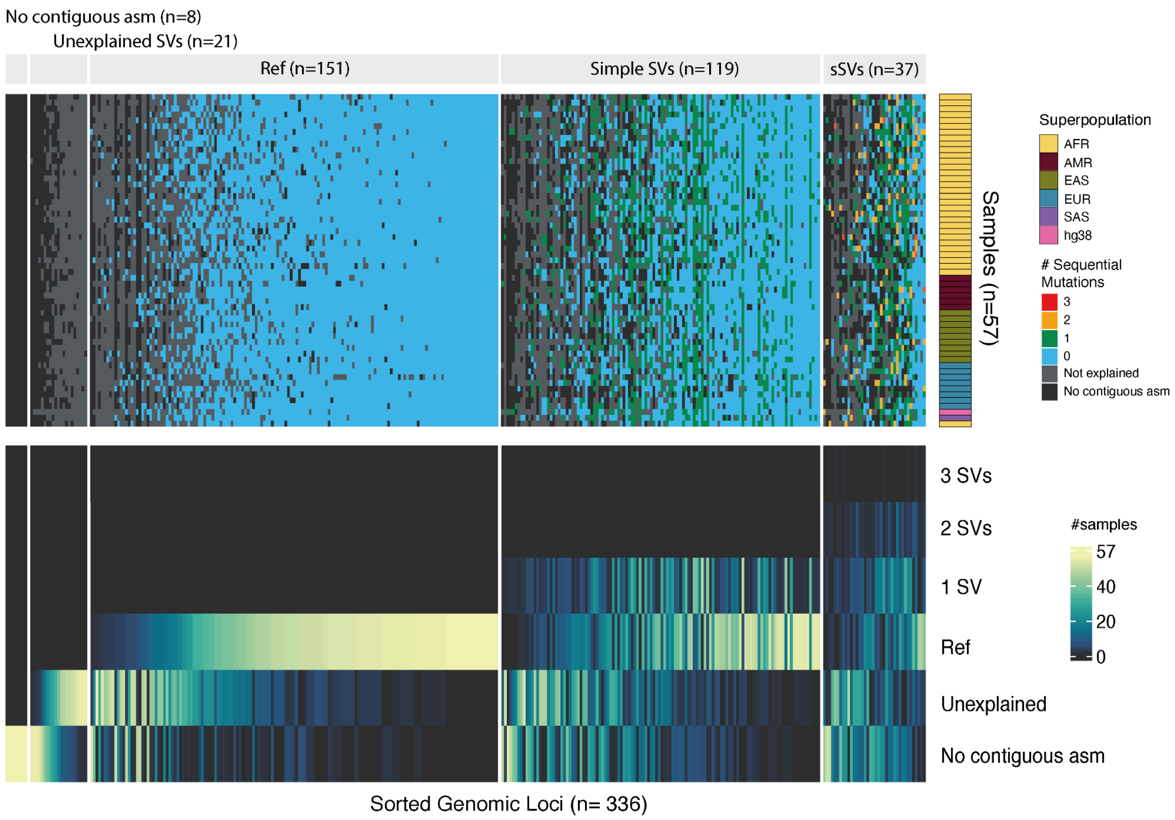

Figure S8. Detailed results of scanning 336 loci across 57 haplotypes with NAHRwhals. (Top) Visualization of every SV call per sample and locus. Loci were grouped according to the number of samples displaying No contiguous assembly, Unexplained SVs and mutations of various depth. Sample ancestry is indicated on the right. **(Bottom)** Simplified view representing the number of various results per locus. n=37 loci displayed nested SVs ('2 SVs', '3SVs') in at least one sample. In cases where loci contained >1 non-overlapping simple SVs, these were reported as '1 SV', reflective of their maximum depth.

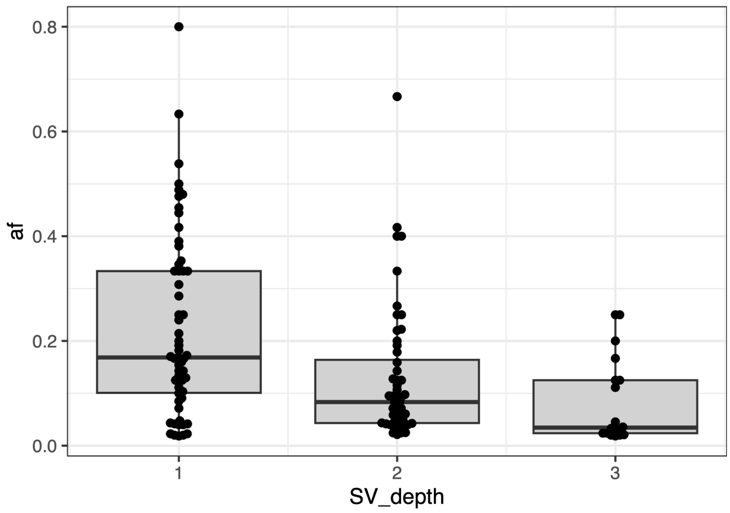

Figure S9. Boxplot showing the correlation between the ‘depth’ of an SV (e.g., ‘inv’ = depth1, ‘inv+del’ = depth2, etc.) and its allele frequency (af). SVs can be seen to get progressively rarer with increasing depth.

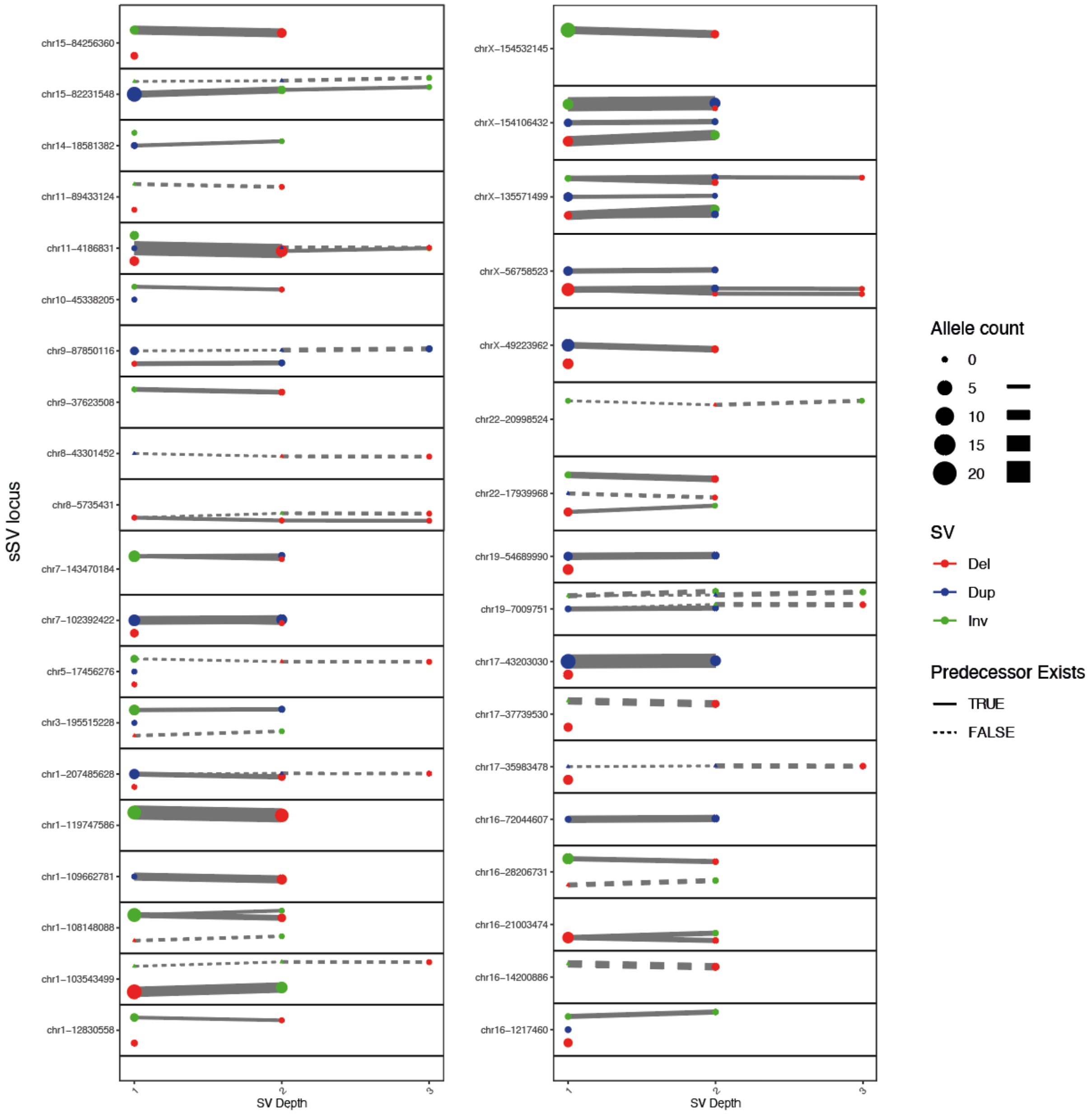

Figure S10. Overview over the predicted relationships between haplotypes, including intermediate states which were predicted by not observed. Each box contains the full set of predicted haplotypes for that locus. Point size indicates the allele count, and the thickness of connections is scaled with the allele count of the downstream SV and is meant to indicate the inferred frequency with which certain ‘transitions’ (such as inv -> inv+del) are likely to arise. Branches starting from haplotypes which are inferred but not observed are denoted with dotted lines. The color of dots corresponds to the SV at that depth.

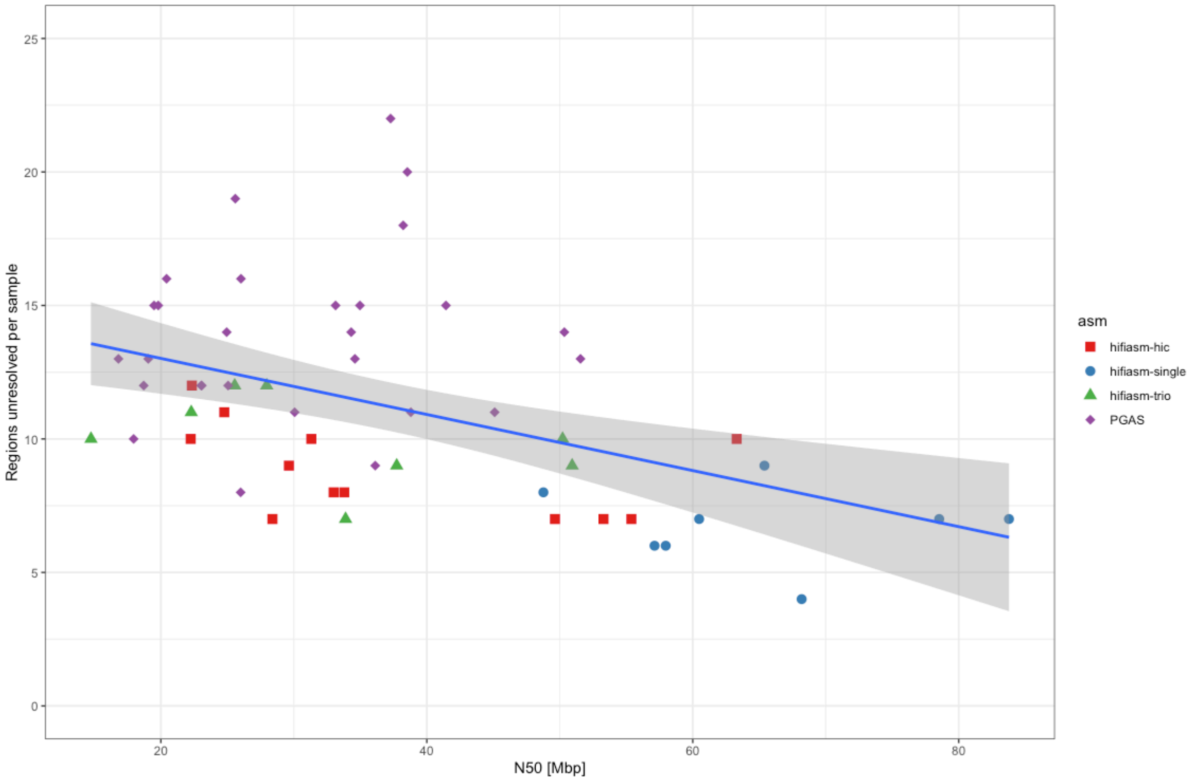

Figure S11. Scatterplot of the overall assembly contiguity measured by the N50 parameter plotted against the number of unresolved regions per sample.

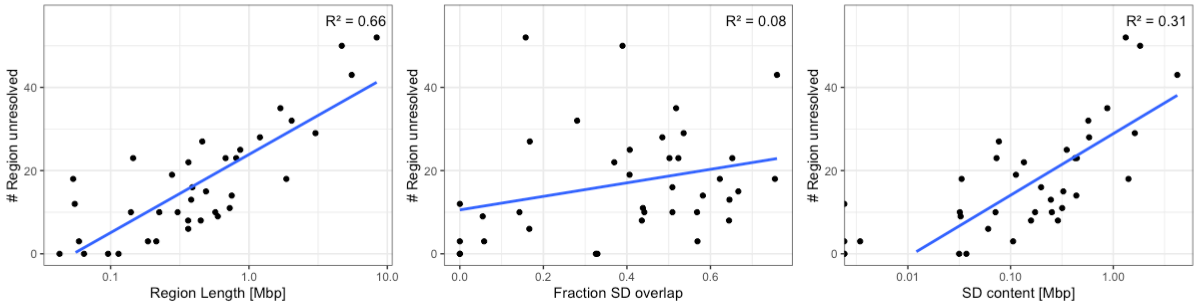

Figure S12. Scatterplots showing the relationship between the number of unresolved regions with the length of a region (left), the fraction of a sequence covered in Segmental Duplications (middle) and the total amount of bases covered in SDs (right).

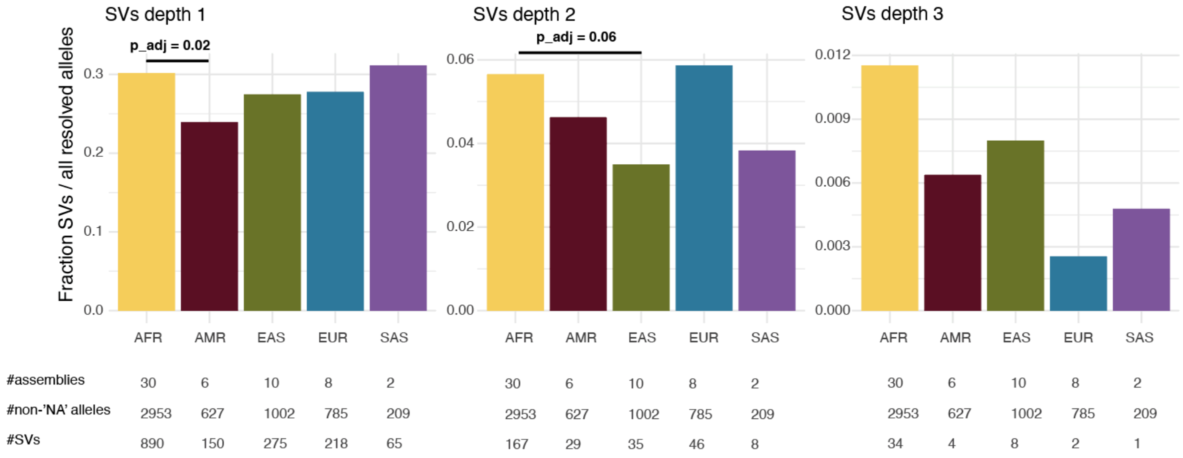

Figure S13. Population-specific numbers of depth 1, 2 and 3 SVs relative to the number of genotyped loci. AFR samples show a slight but significant enrichment in depth-1 and depth-2 SVs compared to samples of admixed american (AMR) and east asian (EAS) ancestry, respectively. However, the low number of samples per superpopulation (AFR: 15, AMR: 3, EUR: 4, EAS: 5, SAS: 1) discouraged further ancestry-based analyses.

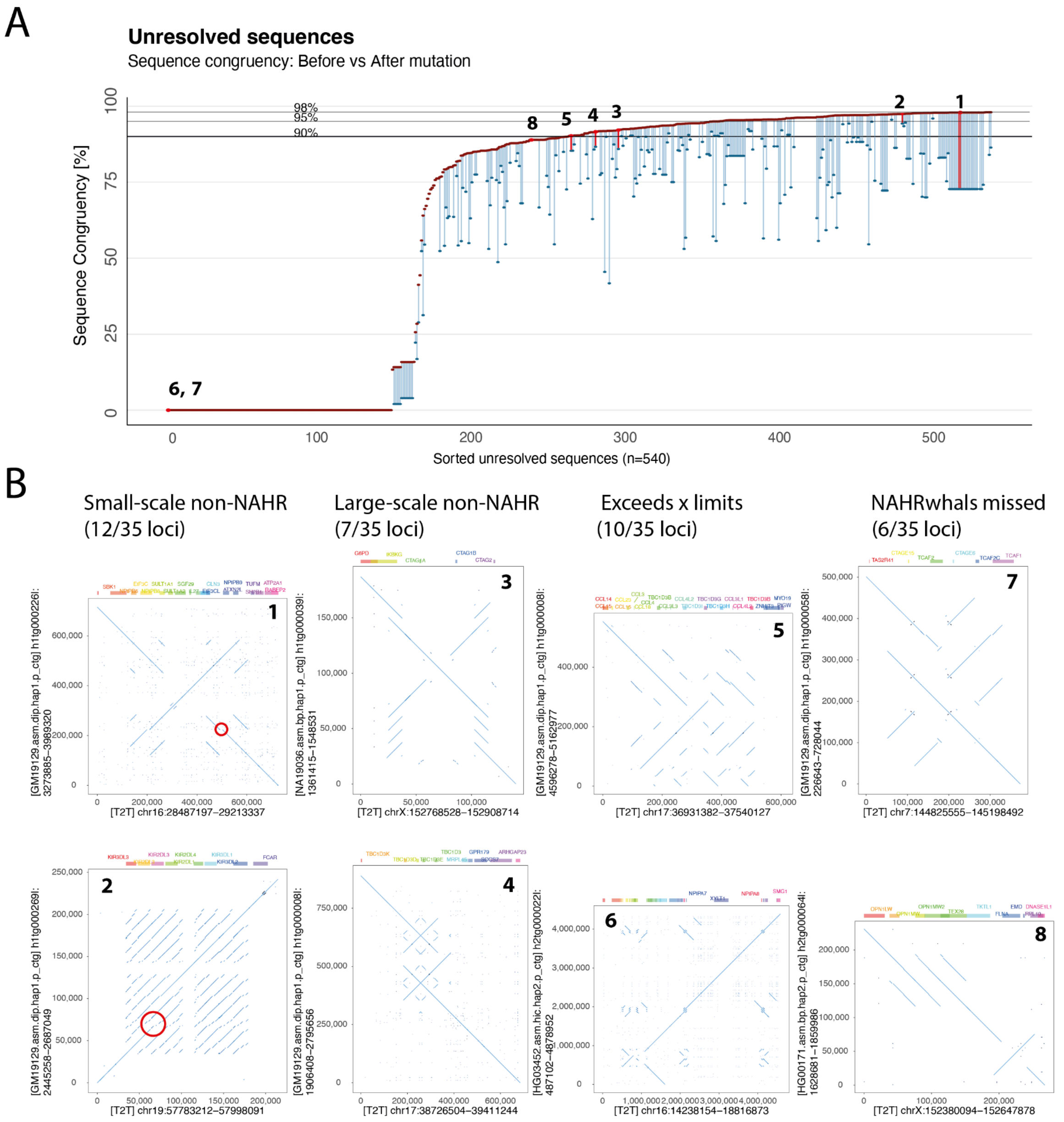

Figure S14. Investigation of variant loci flagged as ‘unexplained’ by NAHRwhals. A A dumbbell plot showing the sequence congruency between each of the 540 unexplained sequences and the CHM13-T2T reference before (blue dots) and after (red dots) application of the highest-scoring mutation chain determined by NAHRwhals. Numerals indicate alignments followed up in panel B. **B** Examples of unexplained alignments determined by manual investigation of a random subset of sequences depicted in A. Unexplained loci group into small-scale non-NAHR events, large-scale non-NAHR events, Mutations exceeding our chosen reference locus and NAHR-mutations which were missed by NAHRwhals.

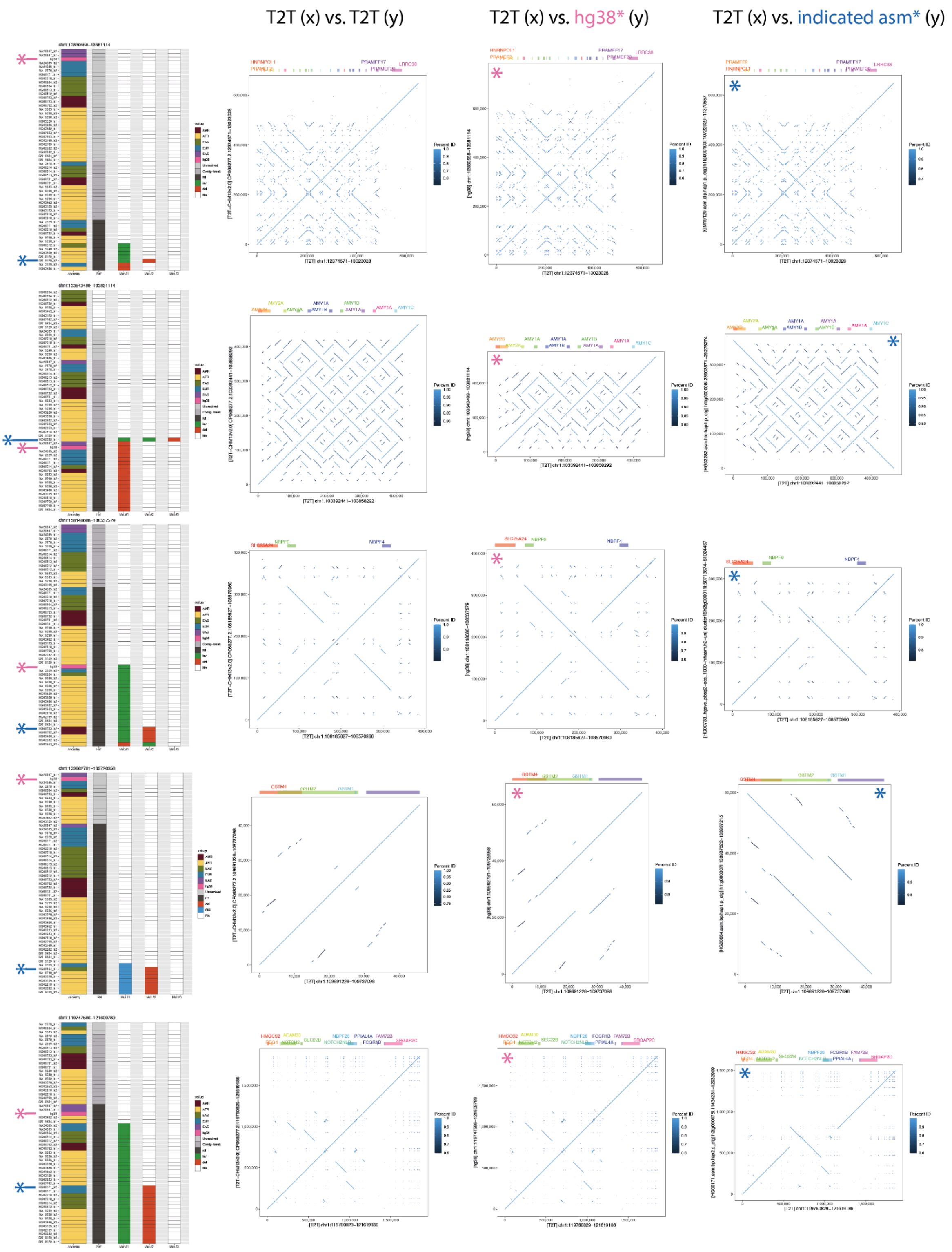

Figure S15. sSV calls and dotplot visualizations of the first 5/37 human sSV loci by start coordinate. For each locus, we display 1) the full SV callset (left), 2) the CHM13-T2T reference aligned to itself, 3) the CHM13-T2T reference aligned to hg38, 4) the CHM13-T2T reference aligned

to the human assembly with the longest called mutational chain. If multiple chains have similar length, we default to displaying the sample with the highest y-value in the sSV-calls plot (left). Colored asterisks are overlaid for visual guidance between genotypes and dot plots. Assembly contigs are, by chance, denoted as the reverse complement to the CHM13-T2T reference in approx. 50% of cases (here: loci 1-4). The directionality of the y axis in the dotplots is thus arbitrary. High-resolution versions of Figures S15-S22 and S30-S32 are available under https://doi.org/10.5281/zenodo.13107026

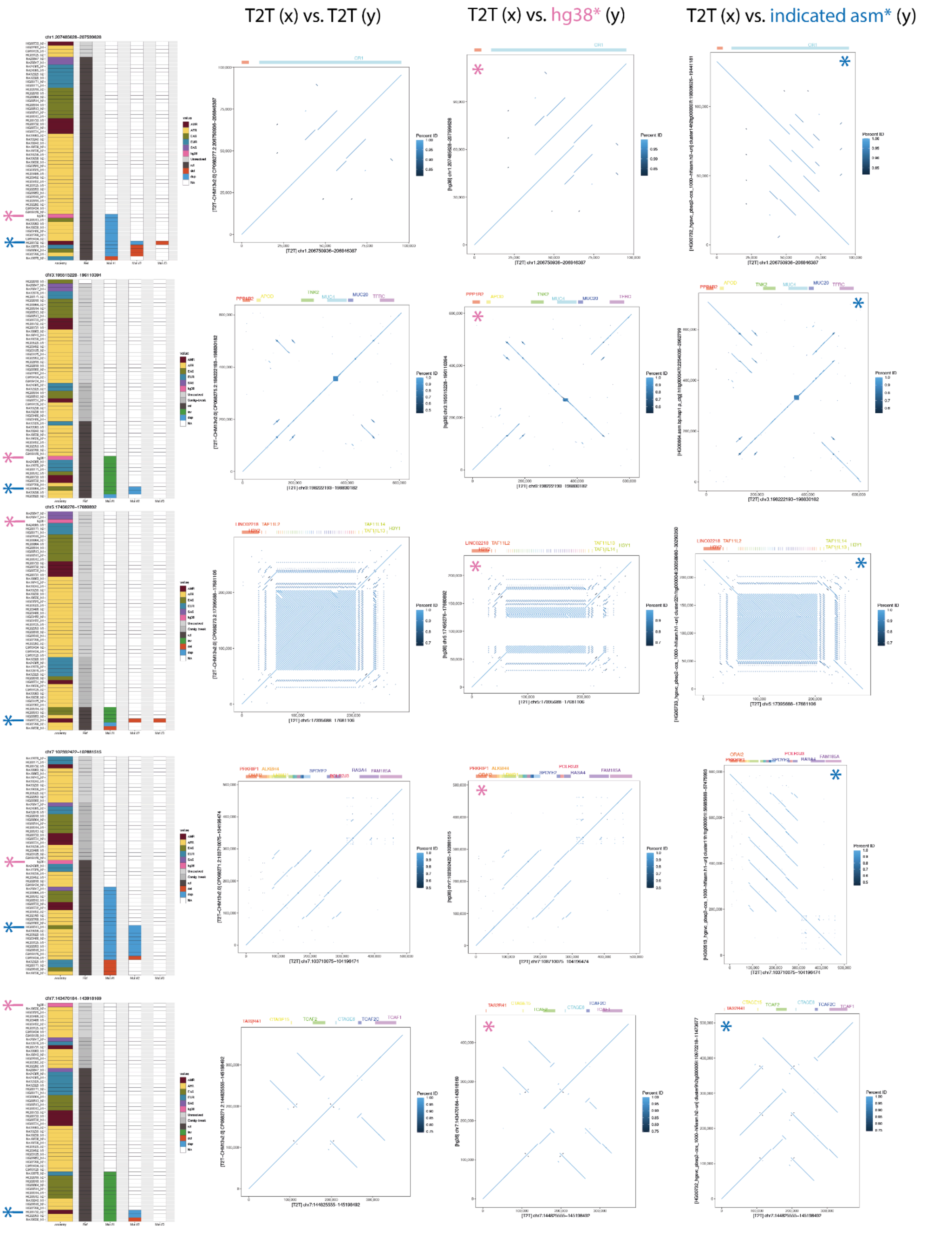

Figure S16. Continuation of **Figure S15.**

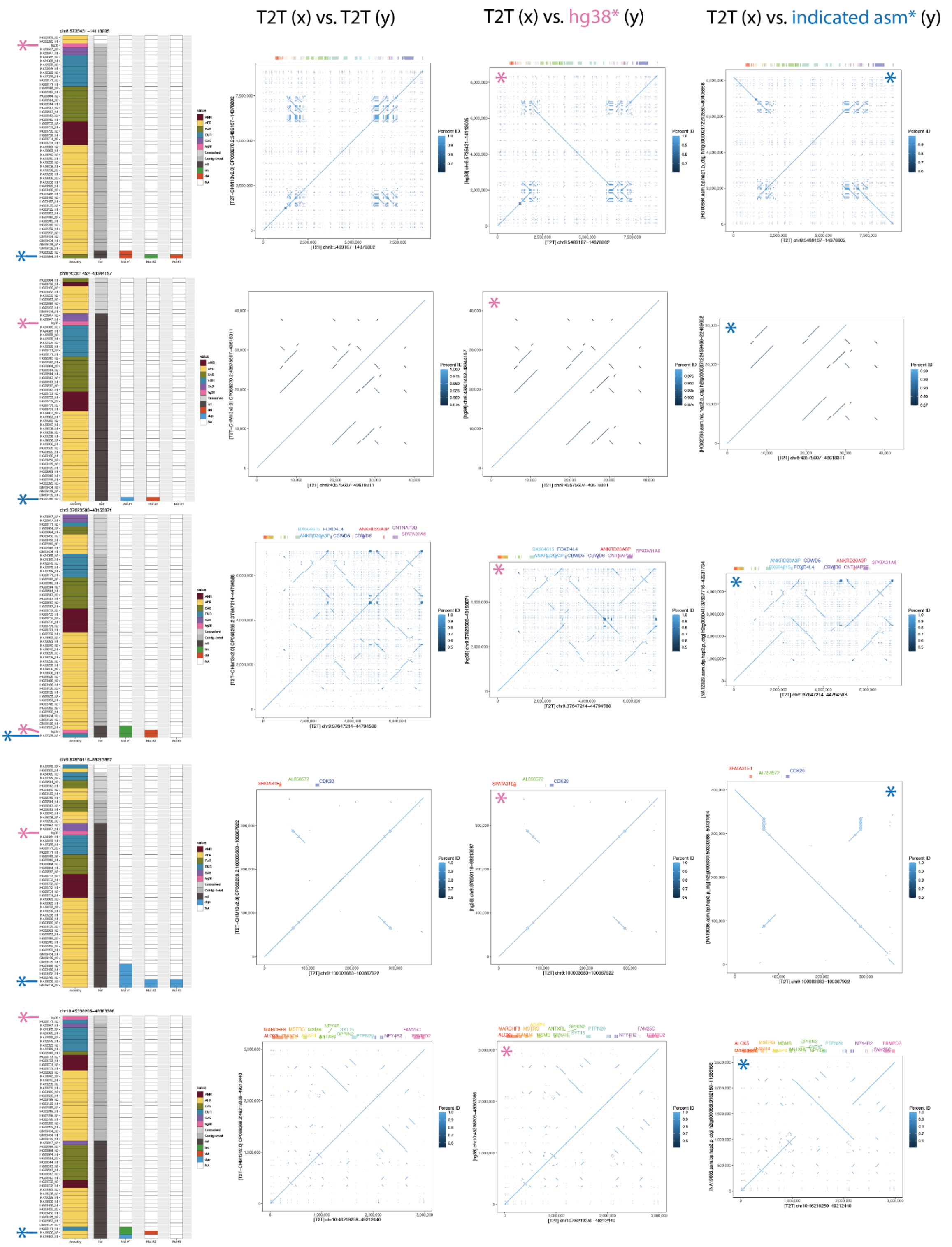

Figure S17. Continuation of **Figure S16.**

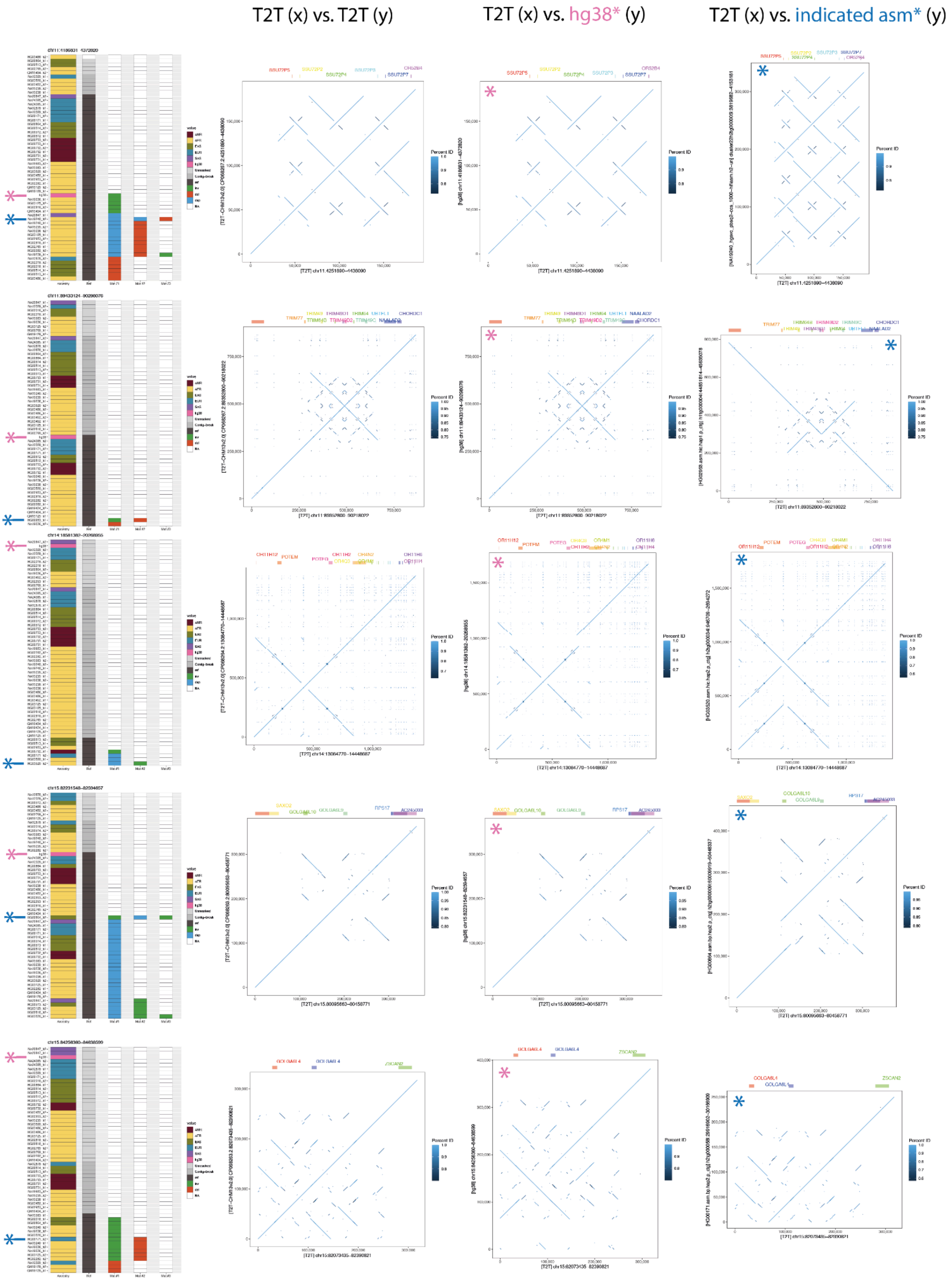

Figure S18. Continuation of **Figure S17.**

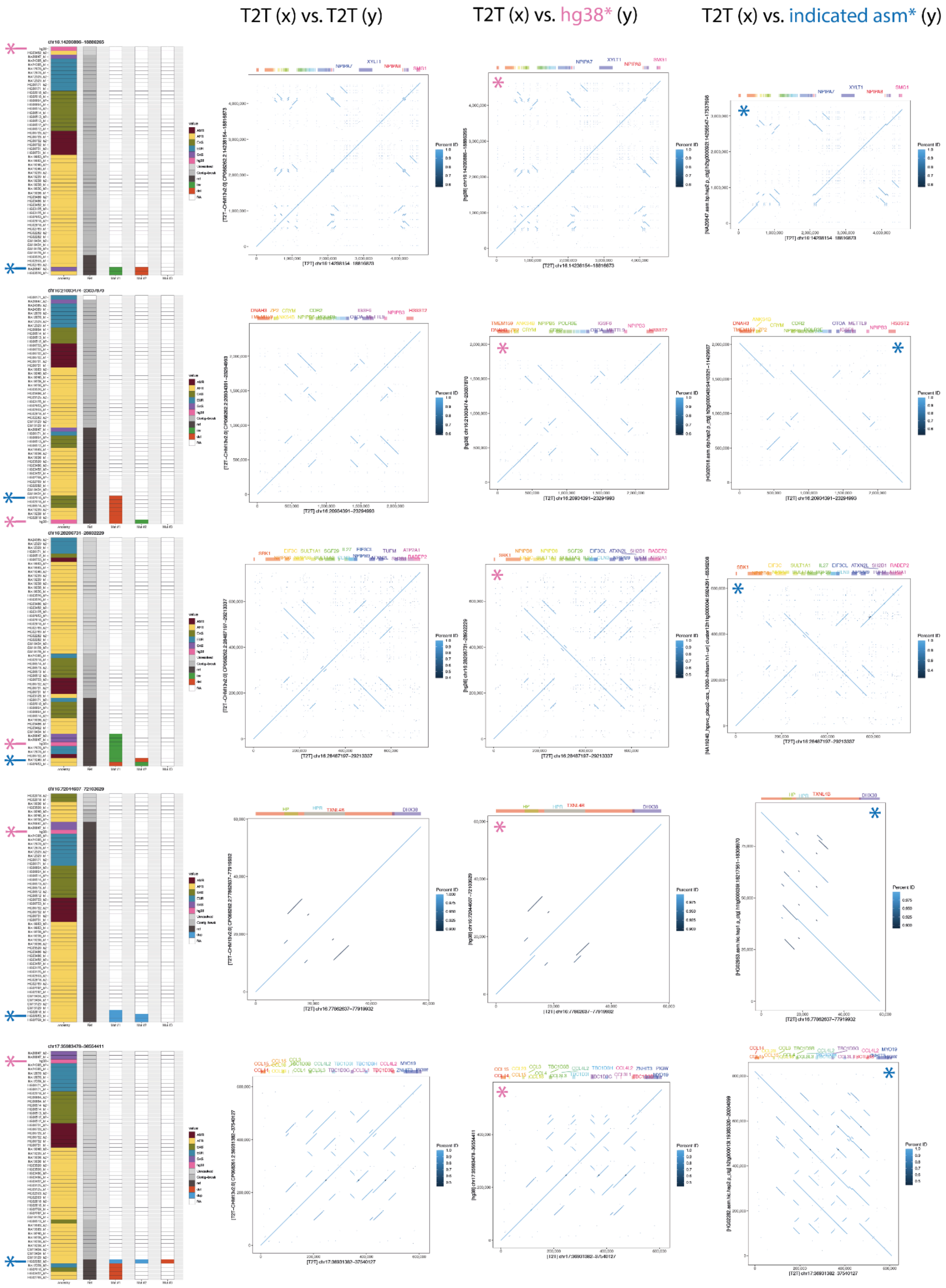

Figure S19. Continuation of **Figure S18.**

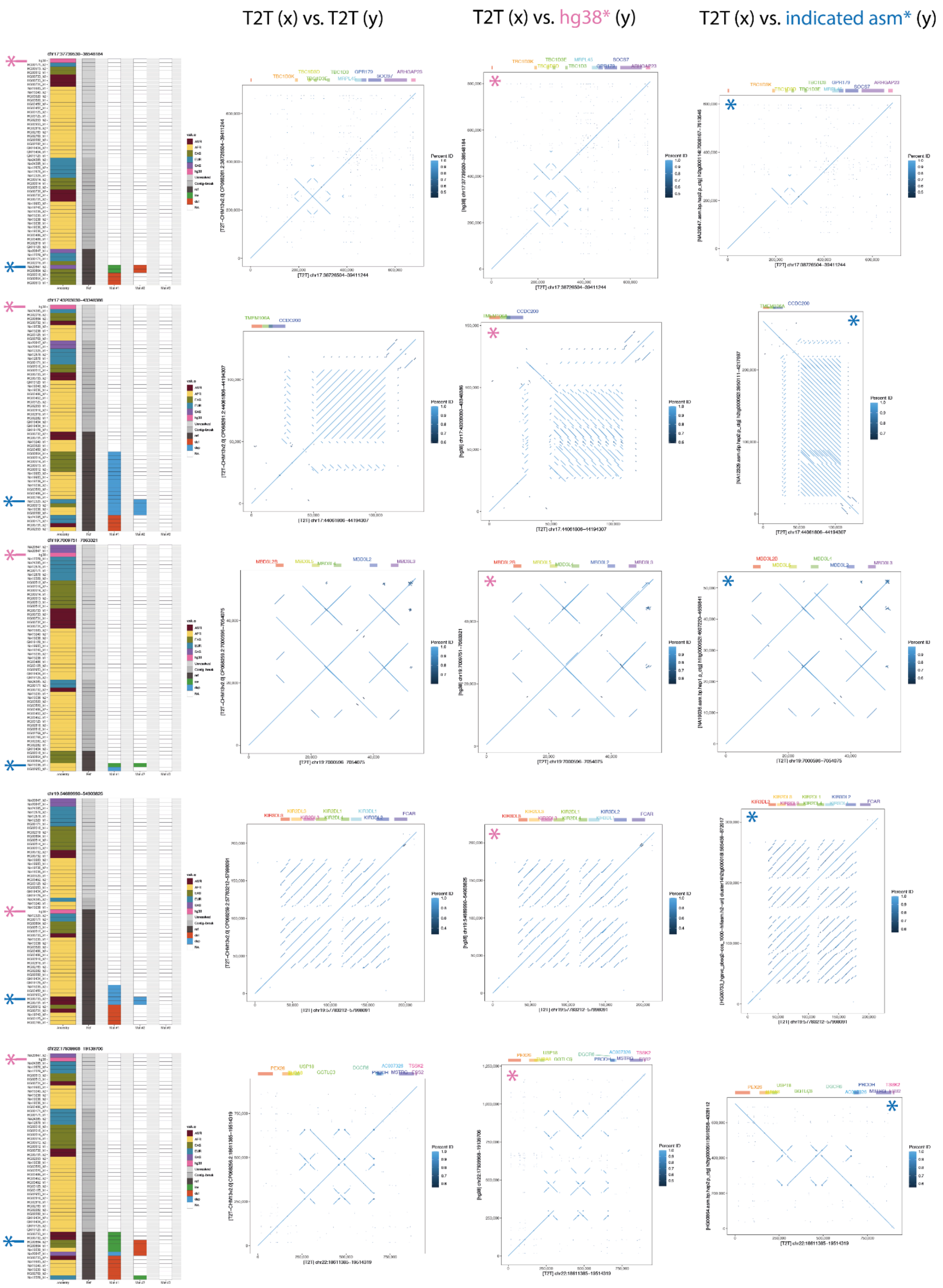

Figure S20. Continuation of **Figure S19.**

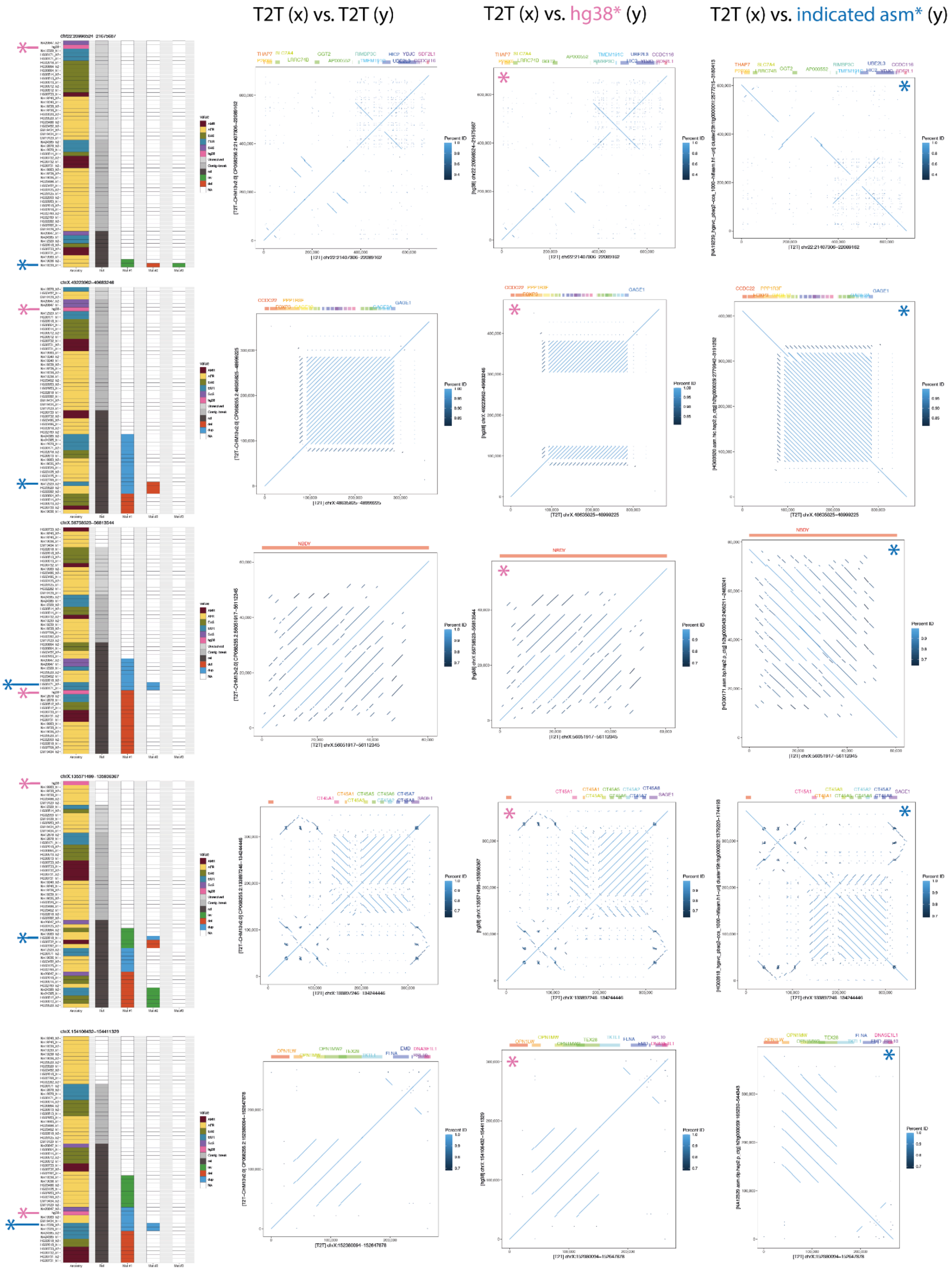

Figure S21. Continuation of **Figure S20.**

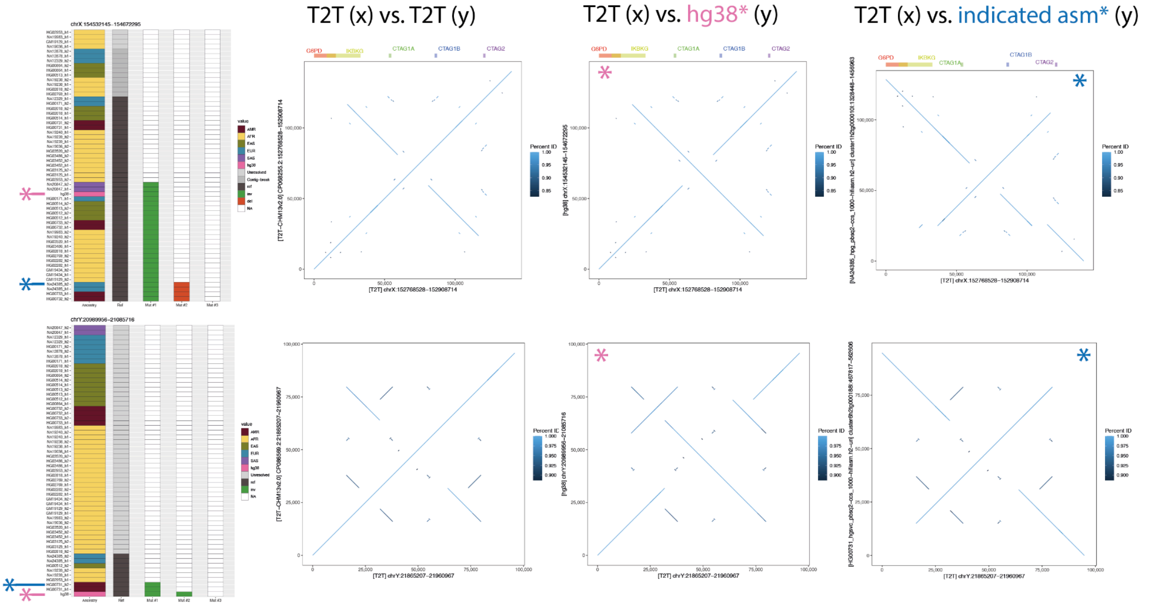

Figure S22. Continuation of **Figure S21.**

A

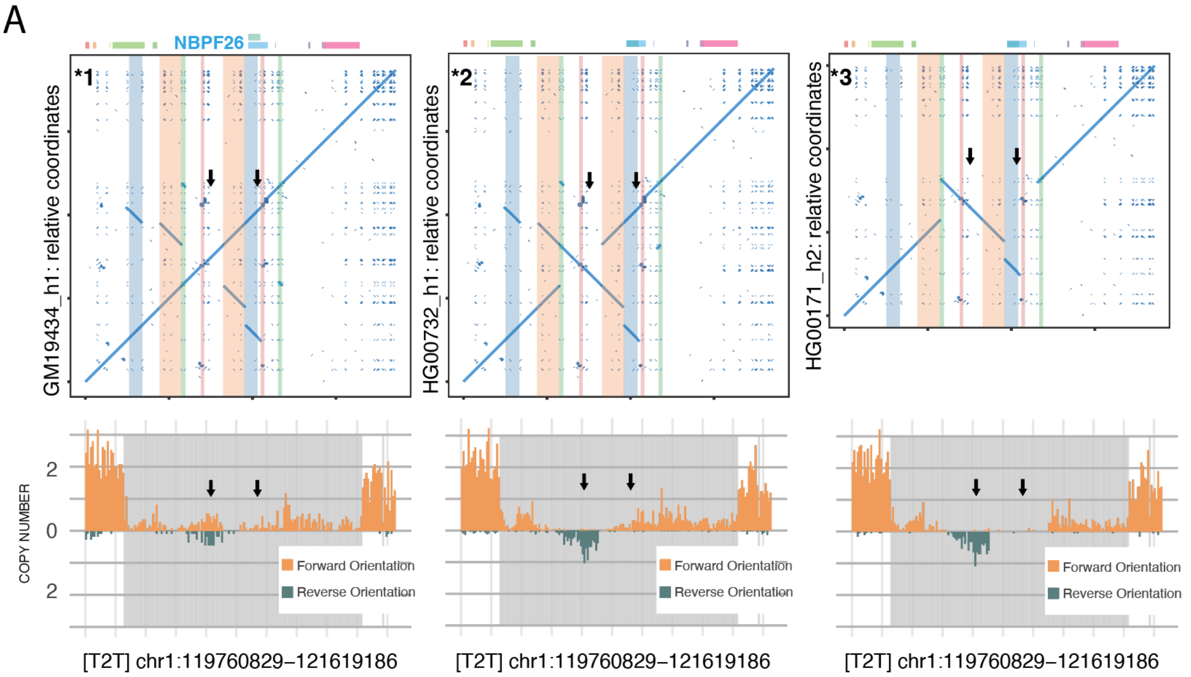

B

**Inversion**		NW calls				
		0/0	0/1	1/1	1/.	./.
SSeq	0/0	1	0	0	0	0
	0/1	0	1	0	0	3
	1/1	0	0	11	3	1
	Sample Missing	0	2	5	1	0

**Deletion**		NW calls			
		0/0	0/1	1/1	./.
SSeq	0/0 or 0/1	5	5	0	7
	1/1	0	0	3	0
	Sample Missing	2	4	1	1

Figure S23. Strand-Seq based validation of chr1:119760829-121619186 INV-DEL sSVs. **A** Dotplot views of the ref, inv and inv+del configurations observed in the locus. Strand-Seq read counts aggregated over single cells (so-called composite-files) are indicated for the same samples below,

confirming a heterozygous inversion (left), homozygous inversion (middle) and a homozygous inversion with deletion (right) in the three samples, respectively. **B** Confusion matrix of NAHRwhals calls (columns) and the Strand-Seq based arbigent calls regarding the presence of an inversion (left table) and a deletion (right side).

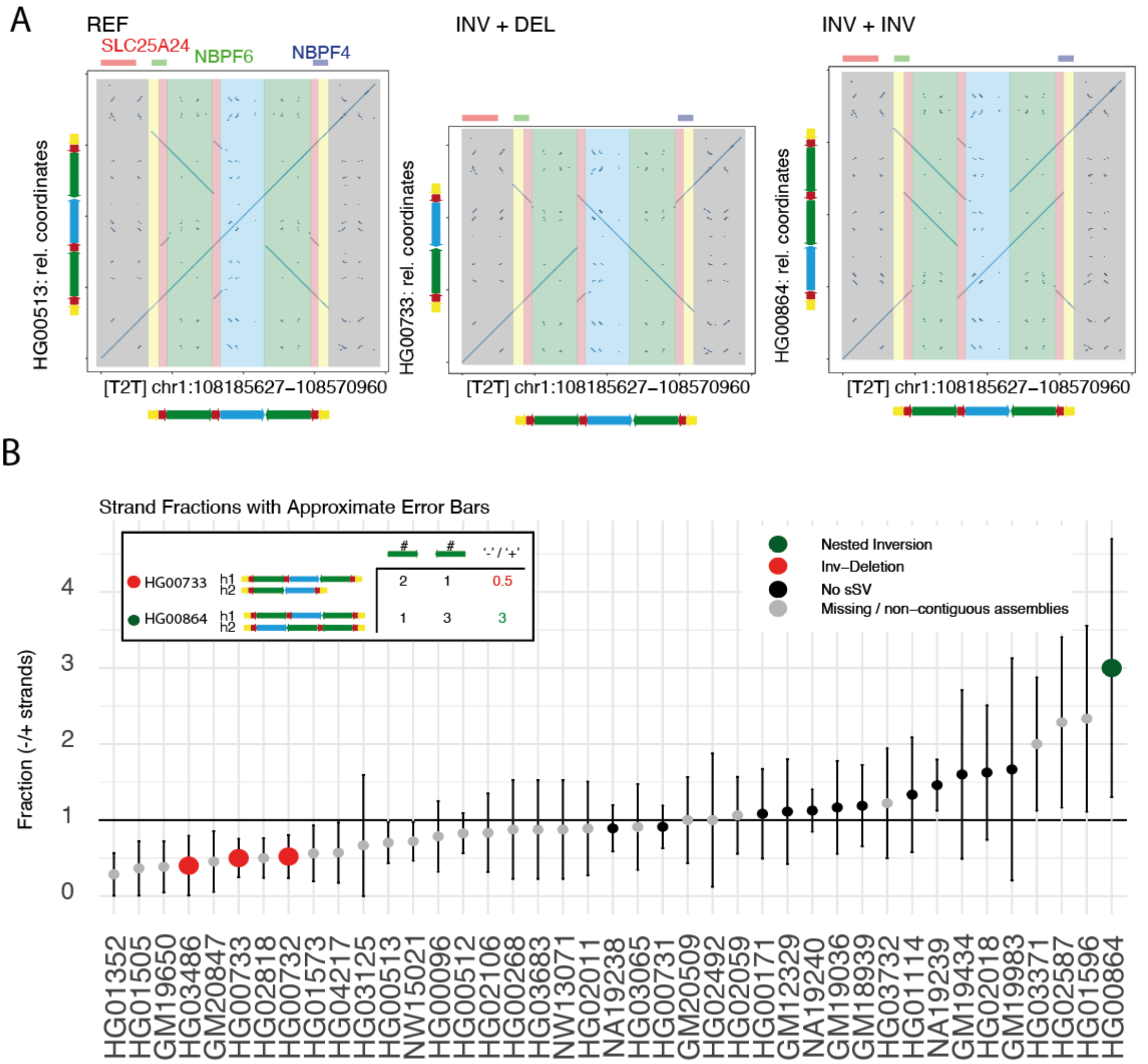

Figure S24. Strand-Seq based validation of chr1:108185627 INV-DEL and INV-INV sSVs. A Dotplot views of three samples showing the Ref, Inv+Del and Inv+Inv states, respectively. **B** With the deletion/duplication being restricted to duplicative regions, we designed a new Strand-Seq based approach in which we count the fraction of reads mapping to the segmental duplication in forward orientation vs reverse orientation. A significant deviation of a 1:1 ratio validates that an SD in forward orientation is missing (i.e. deletion, as in samples HG03486, HG00733, HG00732) or added (as in HG00864). The error bars represent the 95% confidence interval based on binomial sampling uncertainty (methods).

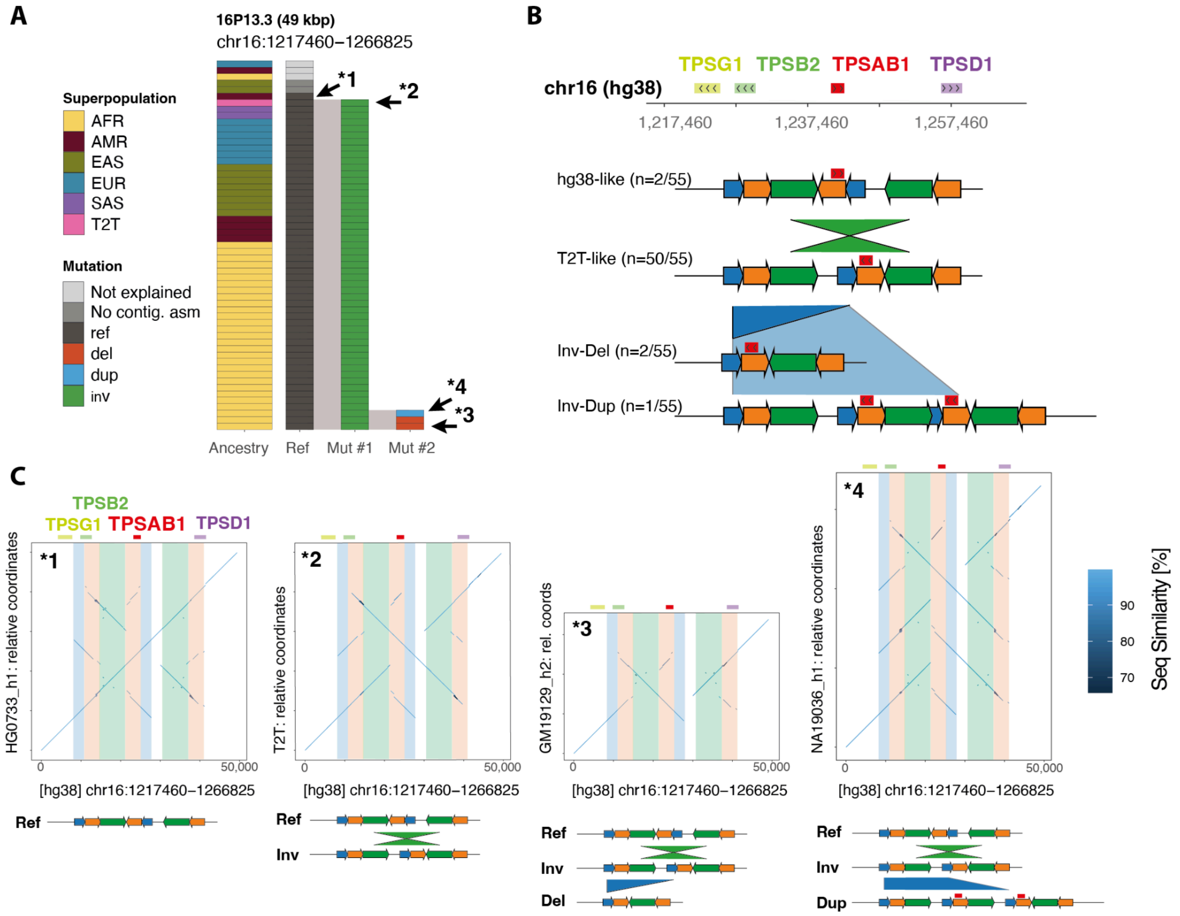

Figure S25. Hg38-exclusive sSVs in the TPSAB1-containing locus. A Mutations identified in the TPSAB1-containing region on chr16p13.3 with respect to the hg38 reference. The majority of haplotypes carry the inverted allele. **B** Schematic of the inferred sSV configurations **C** Dotplots visualising four distinct haplotype configurations, two of which carry potentially functional CNVs.

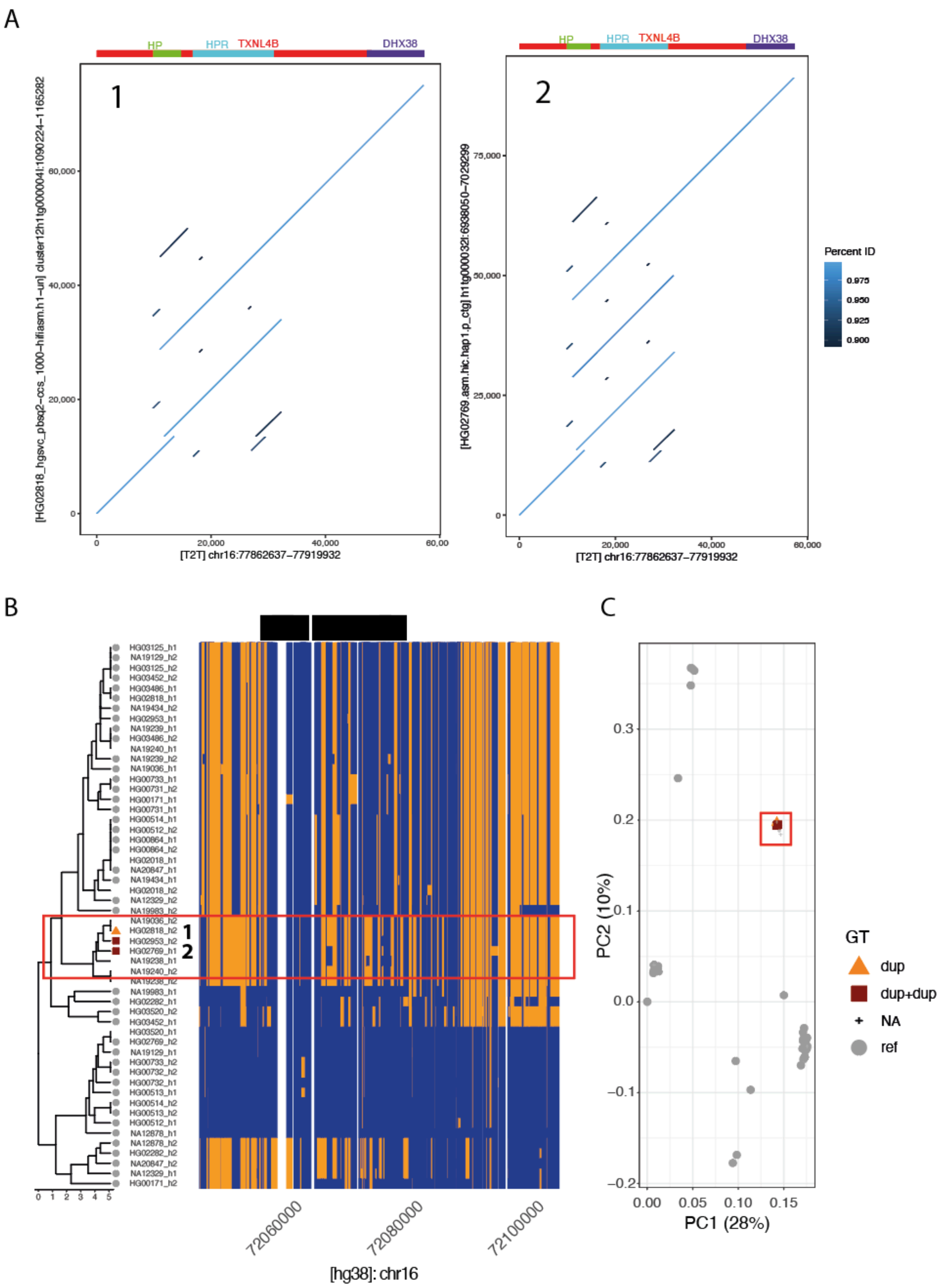

Figure S26. SNP-based population analysis of sSV locus T2T:chr16:77862637-77919932. A dotplot views of the 'dup' and 'dup+dup' configurations observed in the region. **B** the depth-2 'dup-dup' can be seen clustering with its presumed predecessor, 'dup', supporting a serial formation of the dup+dup.

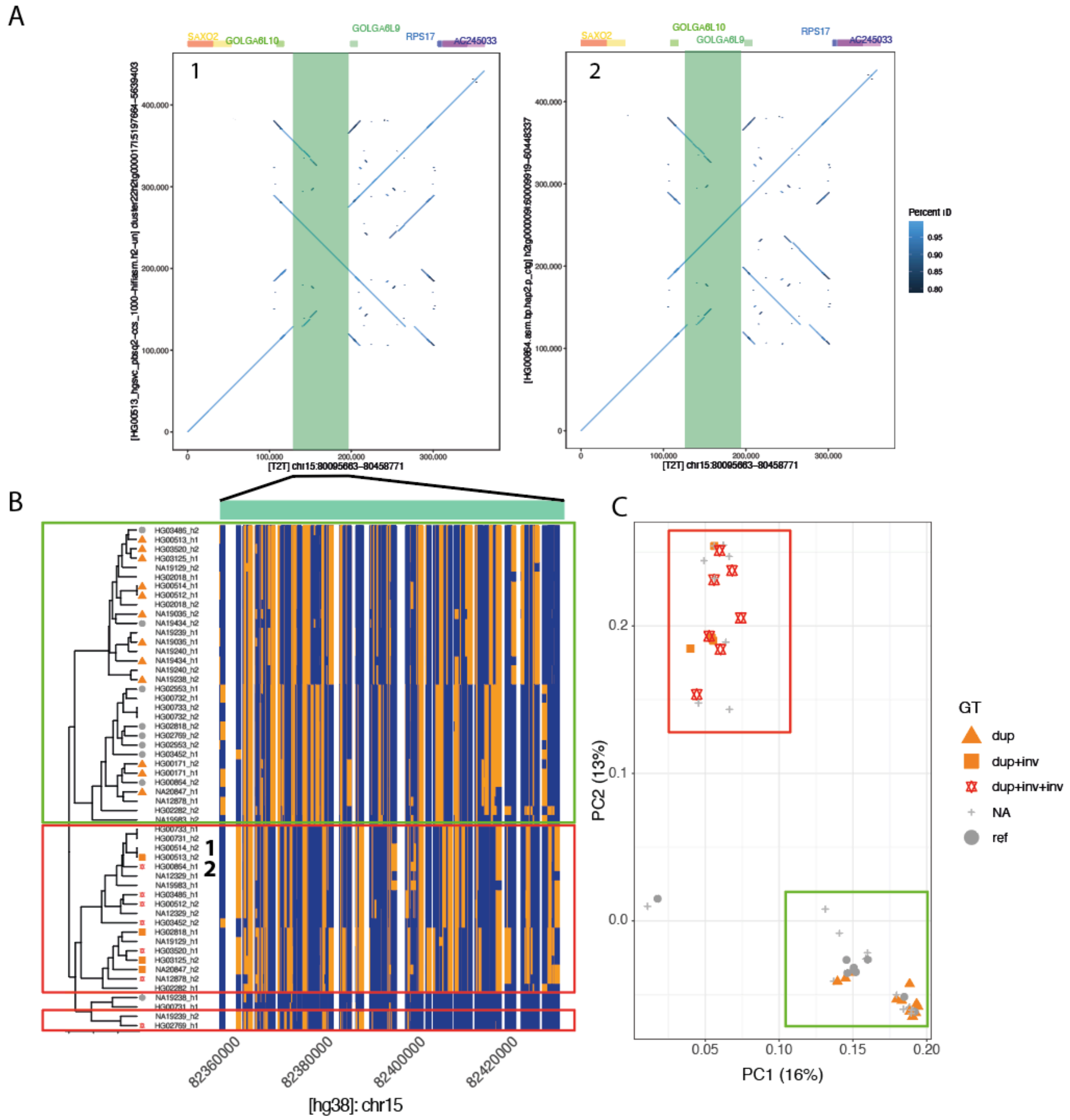

Figure S27. SNP-based population analysis of sSV locus T2T:chr15:80095663-80458771. A dotplot views of the ‘dup+inv’ and ‘dup+inv+inv’ configurations observed in the region. **B** Based on the SNP profile within the inverted, mostly unique region, the haplotypes form two clusters, one containing ref and dup haplotypes, and the other the variants of depth 2 and 3, ‘dup+inv’ and ‘dup+inv+inv’. The apparent intermixing of samples within each cluster suggests a rapid turn-over of architectures (e.g. through a recurrent inversion in the ‘red’ cluster) that generally exceeds SNP formation timescales.

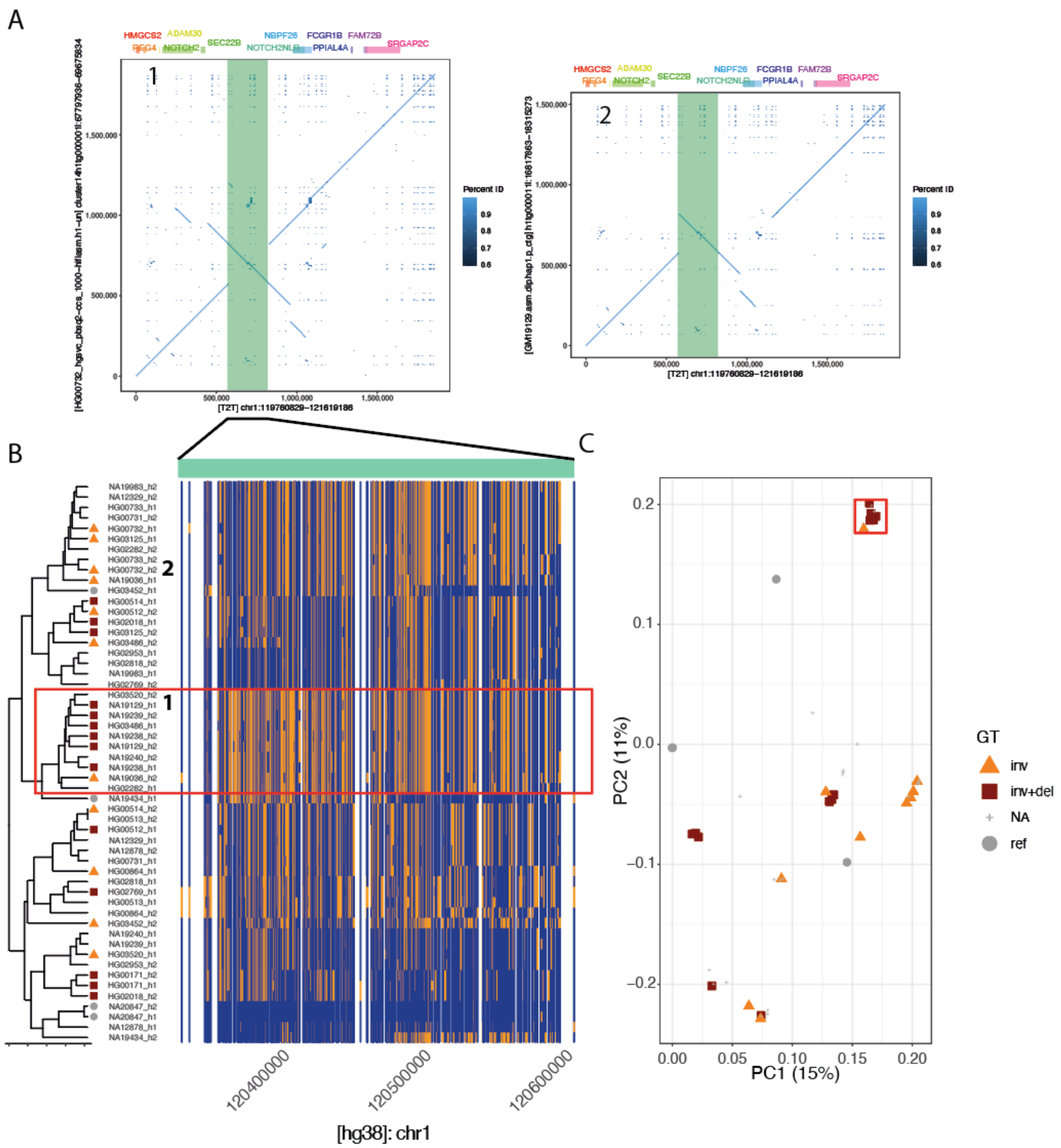

Figure S28. SNP-based population analysis of sSV locus T2T:chr1:119760829-121619186. A dotplot views of the ‘inv’ and ‘inv+del’ configurations observed in the region. **B** Clustering of haplotypes based on SNPs found within the unique region found inside the sSV locus (lifted over to hg38). We highlight a detached cluster of ‘inv+del’ haplotypes (red) which is associated with a single ‘inv’ haplotype (validated in StrandSeq), which might be the predecessor of this group of ‘inv+del’. The genotypes in this region have been orthogonally validated using Strand-Seq.

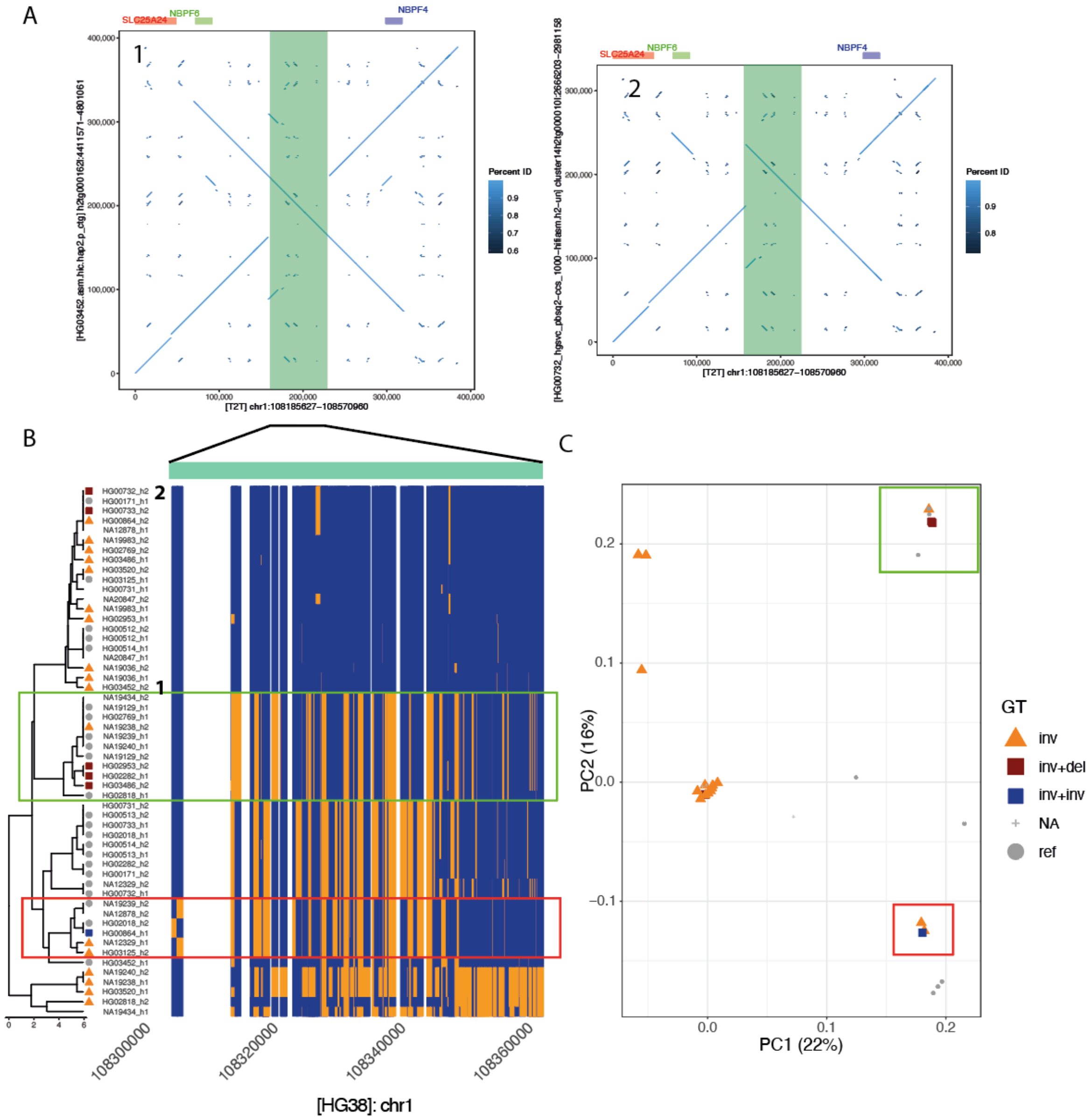

Figure S29. SNP-based population analysis of sSV locus T2T:chr1:108185627-108570980. A dotplot views of the 'inv' and 'inv+del' configurations observed in the region. **B** Clustering of haplotypes based on SNPs found within the unique region found inside the sSV locus. We highlight two clusters of closely related haplotypes, which show a high degree of SV variation. The genotypes in this region have been orthogonally validated using Strand-Seq.

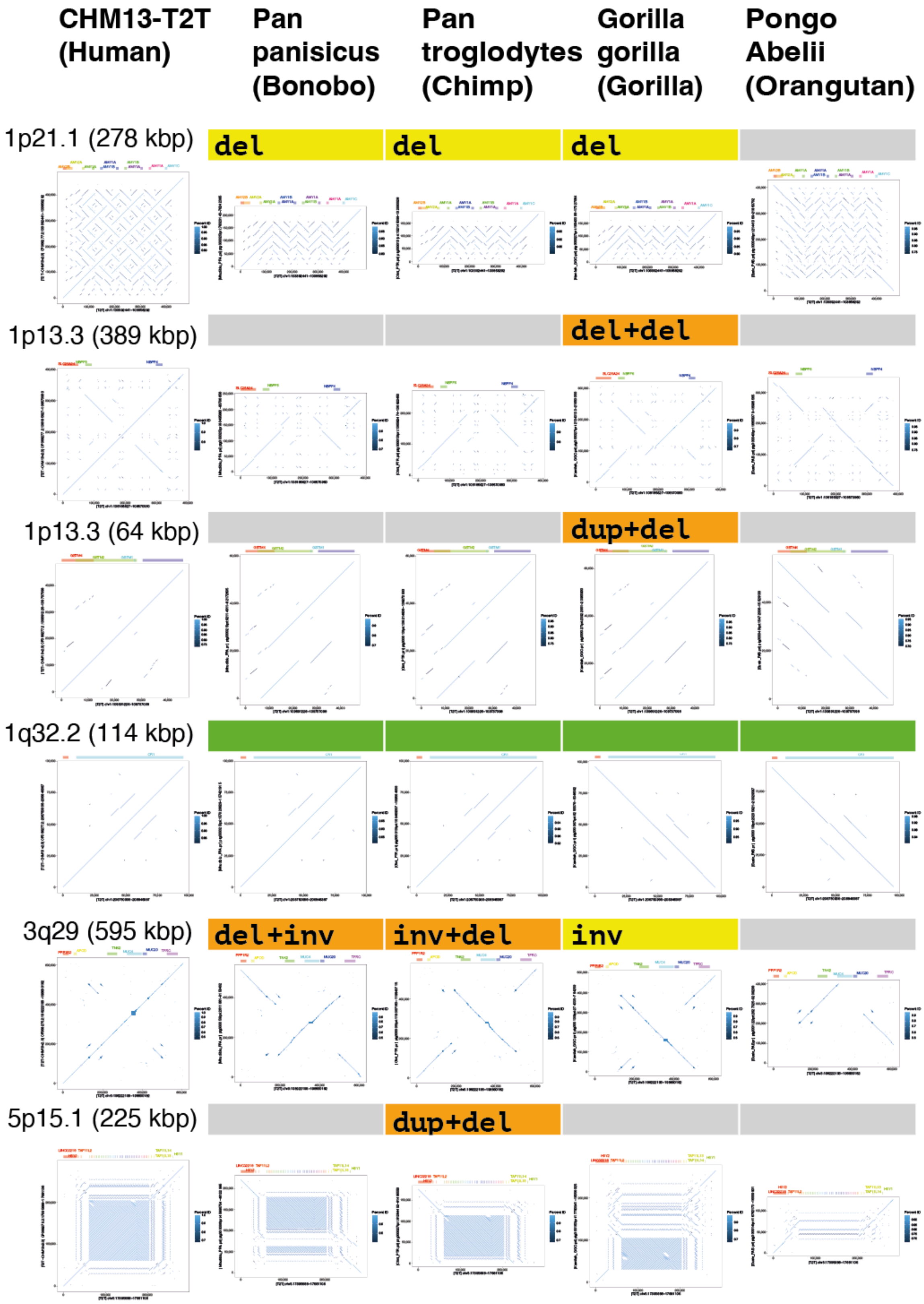

Figure S30. Dotplots of 16 human sSV regions reported as 'resolved' in at least one great ape assembly. Reported genotypes (relative to CHM13-T2T) are indicated above each dotplot. The directionality of the y axis in the dotplots is arbitrary given the random orientation (direct vs reverse

complement) of contigs. Green: reference. Light grey: unexplained variance. High-resolution versions of Figures S15-S22 and S30-S32 are available under https://doi.org/10.5281/zenodo.13107026.

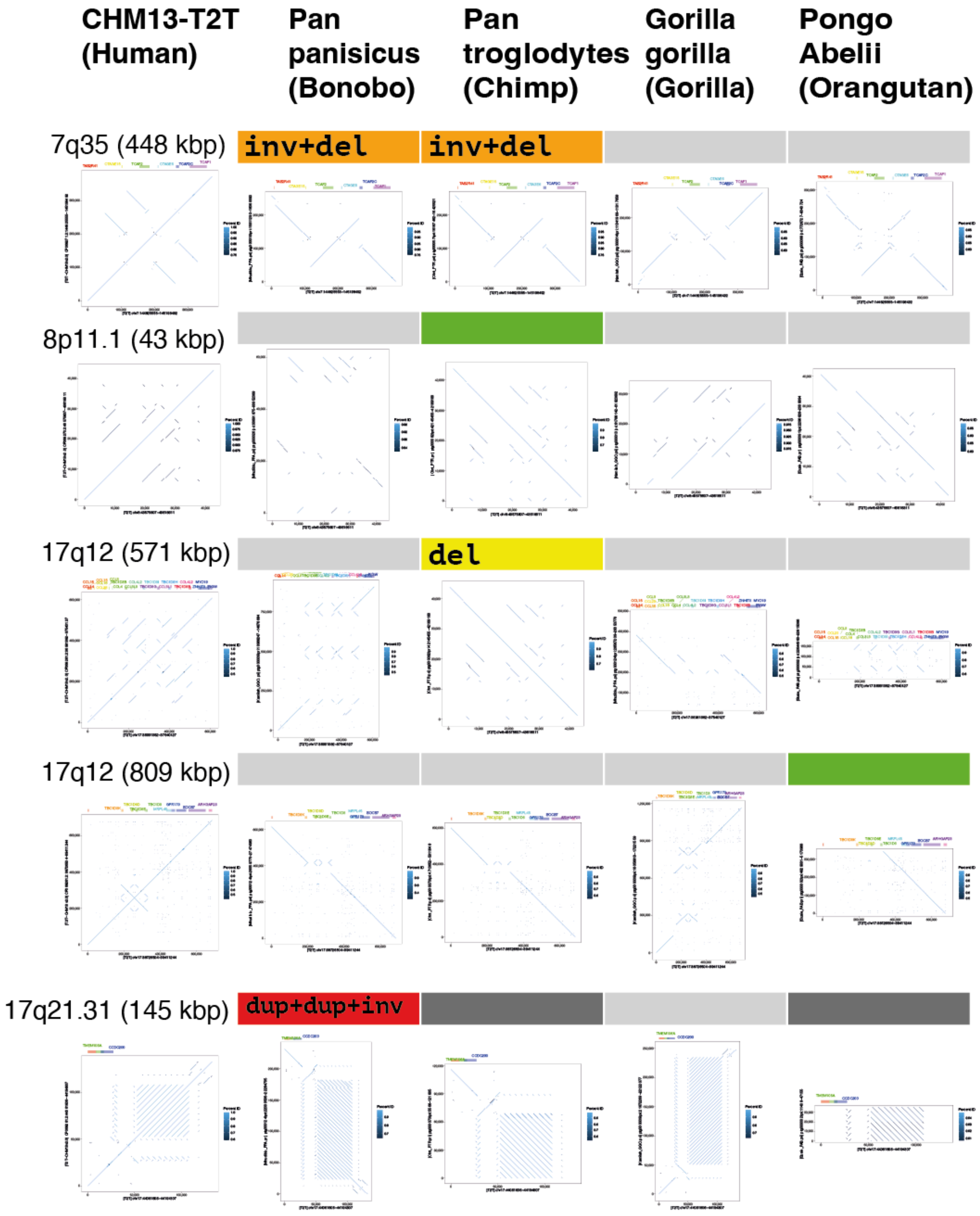

Figure S31. Continuation of Figure S30. Dark grey: non-continuous assembly.

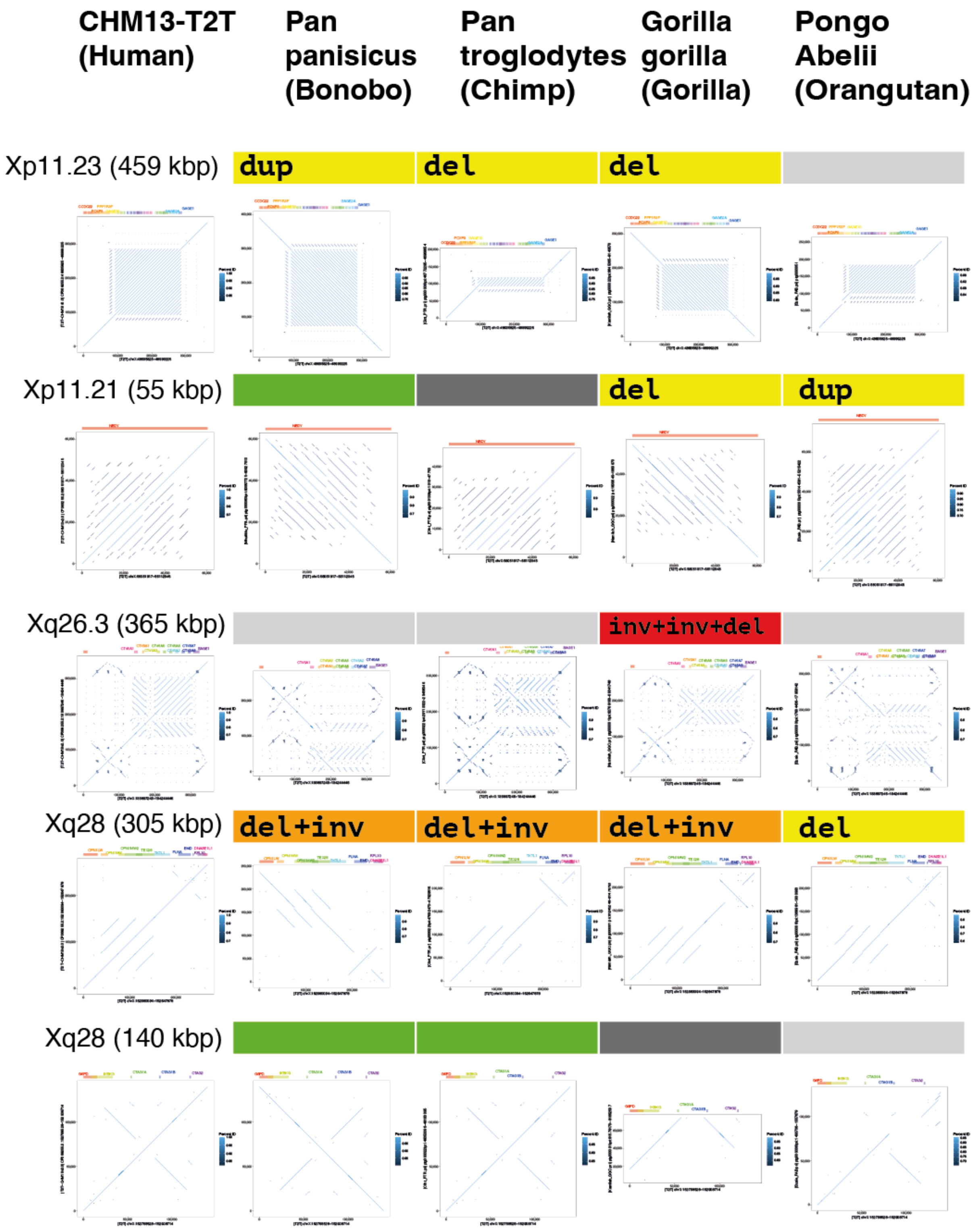

Figure S32. Continuation of Figure S31.

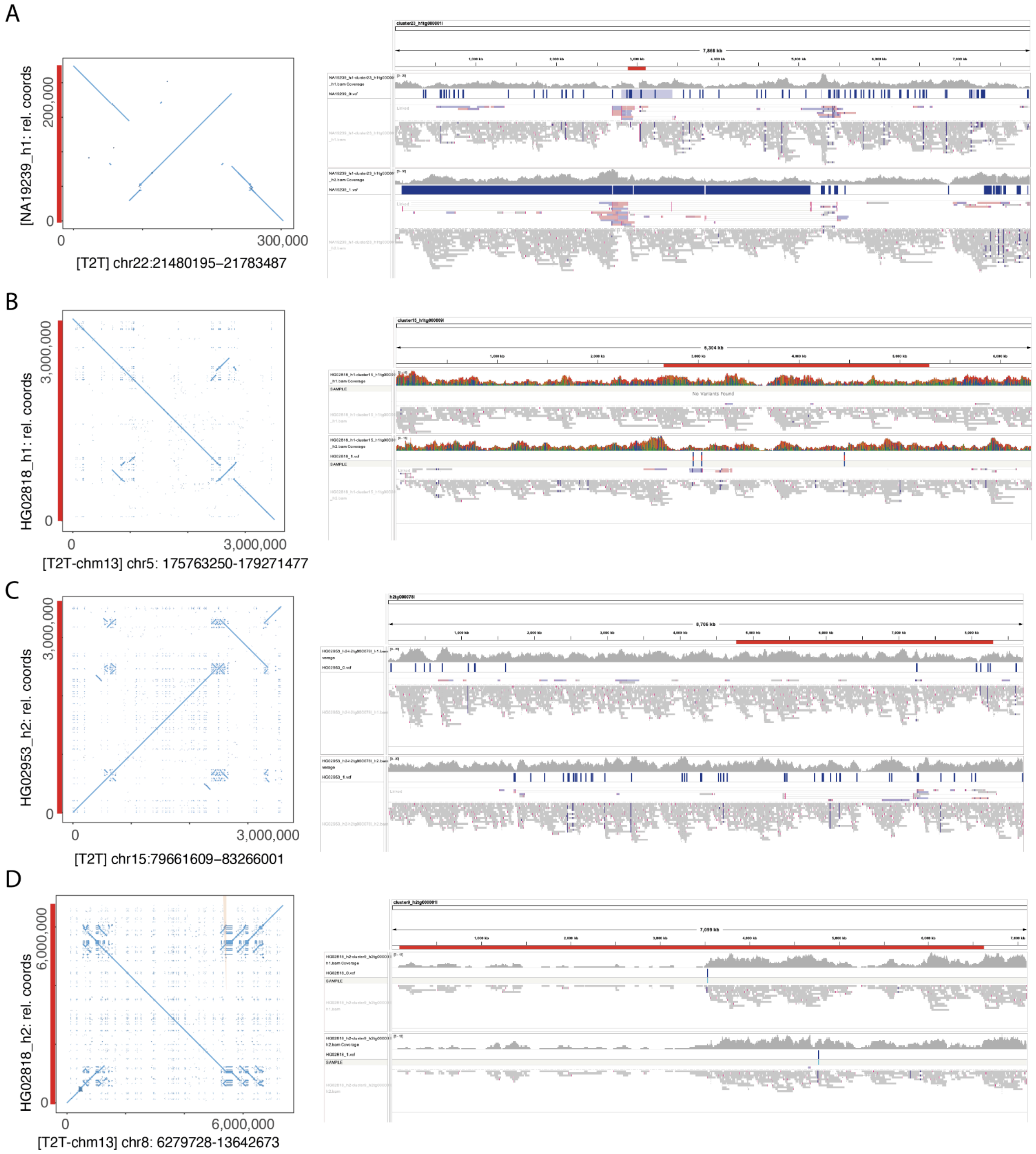

Figure S33. ONT reads aligned to their respective assemblies in four morbid CNV - containing regions. IGV screenshots of nanopore reads mapping to the entire assembled contig are depicted on the right, with a red highlight bar indicating the position of the window region of interest. Sniffles-based calls per haplotype are overlaid over each read track. Reads were split into haplotypes h1 (top track per panel) and h2 (bottom track per panel) using samtools phase. The depicted regions are **A** Di-George syndrome region, **B** Sotos syndrome region, **C** 15q25.2 del/dup region, **D** 8q23.1 del/dup region.

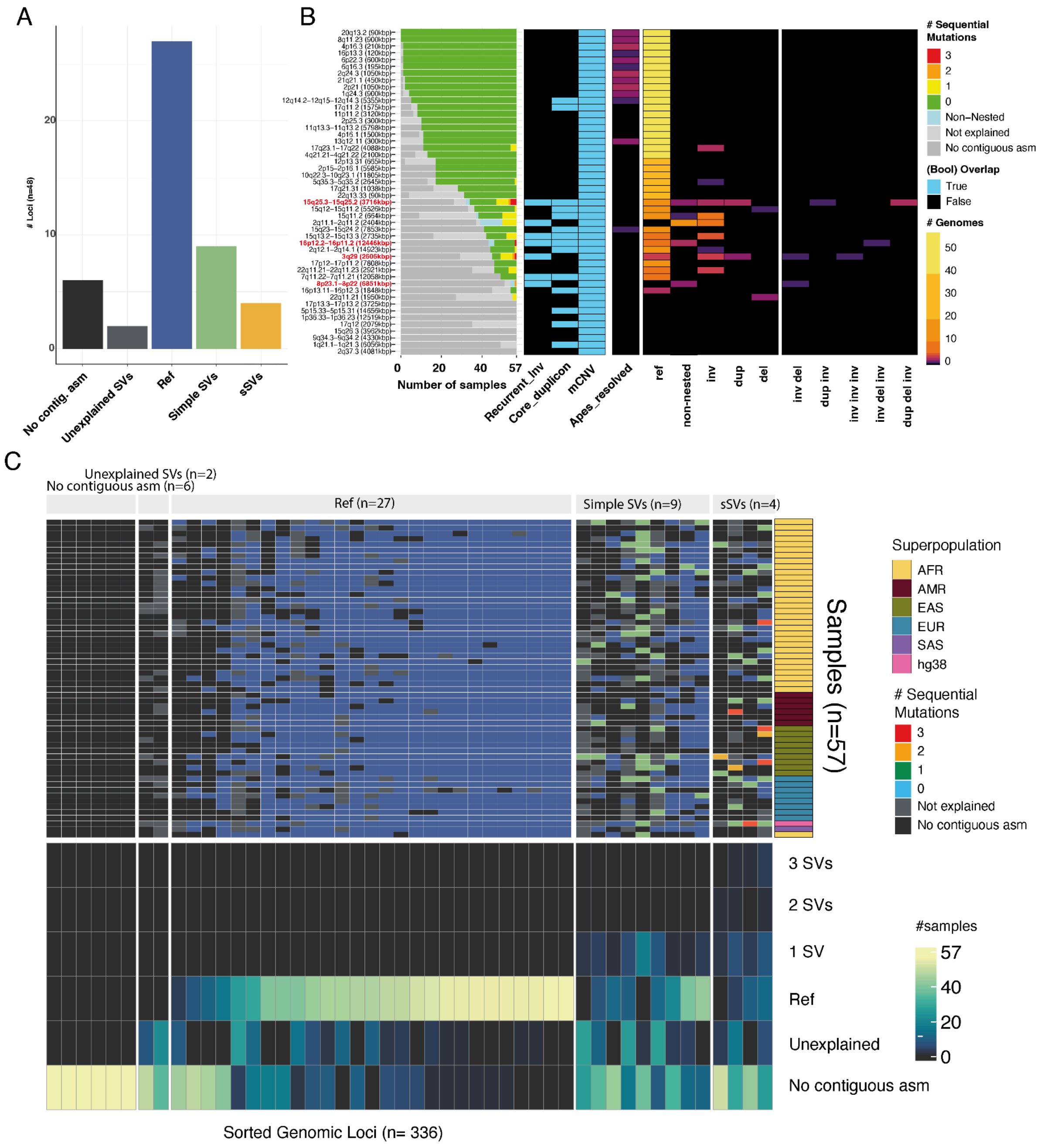

Figure S34. Overview over the NAHRwhals results of scanning 48 mCNV-associated regions for sSV content. A Broad classification of the 48 loci surveyed with NAHRwhals. Loci were considered as ‘sSVs’ if they displayed at least one overlapping pair of SVs in at least one sample. **B** Overview over the full callset of all 48 loci. The diagram shows the prediction performance in humans and apes (‘SVs resolved’), the presence of recurrent inversions, core duplicon-mapping genes and morbid CNV regions in the genomic region, as well as genotypes for each locus. **C (Top)** Visualization of every SV call per sample and locus. Loci were grouped according to the number of samples displaying No contiguous assembly, Unexplained SVs and mutations of various depth. Sample ancestry is indicated on the right. **(Bottom)** Simplified view representing the number of various results per locus. n=37 loci displayed nested SVs (‘2 SVs’, ‘3SVs’) in at least one sample. In cases where loci

contained >1 non-overlapping simple SVs, these were reported as ‘1 SV’, reflective of their maximum depth.

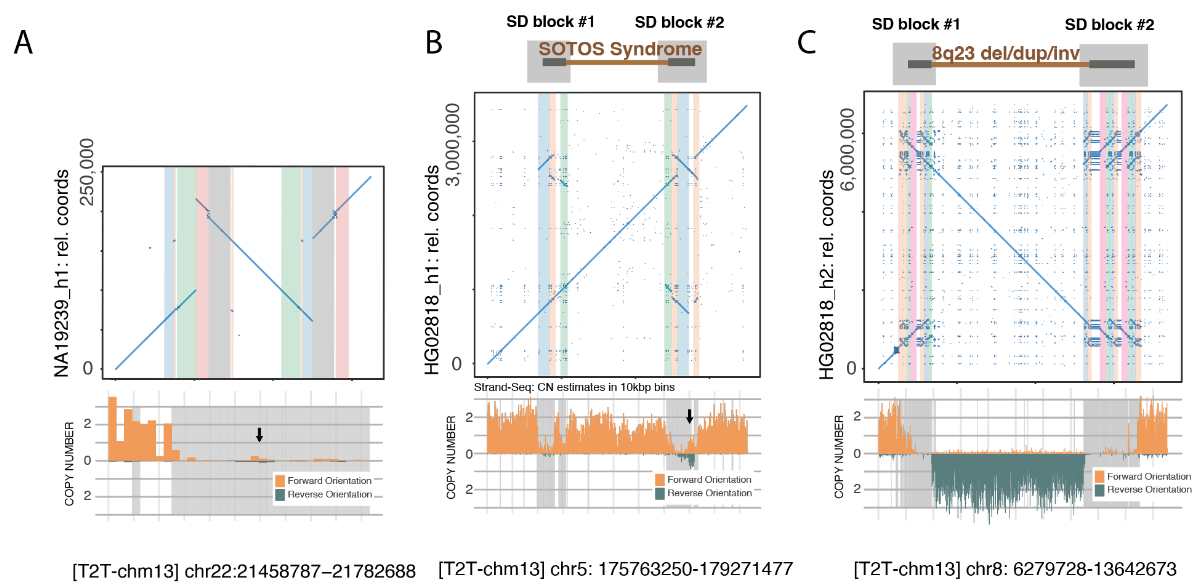

Figure S35. Strand-Seq based validation of three potentially medically relevant sSV loci. **A** The predicted inversion around chr22:21458787 is faintly visible in Strand-Seq. **B** A heterozygous inversion at one end of the SOTOS syndrome region is clearly visible in Strand-Seq. **C** A large, well-known inversion in region chr8p23 is supported by StrandSeq.

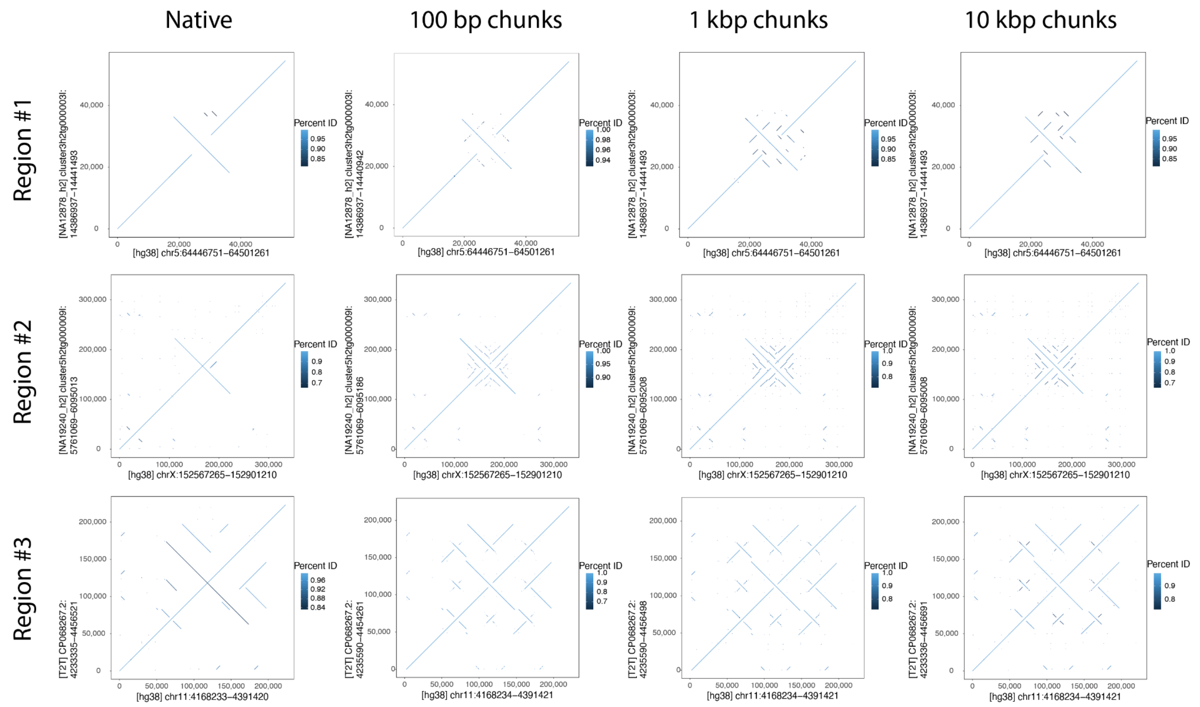

Figure S36. Three example loci illustrating the effect of chunking input query sequences before alignment. All panels were created with minimap2 (version 2.18-r1035-dirty; parameters *-x asm20 -P -c -s 0 -M 0.2).* Without chunking ('Native'), alignments did not resolve all segmental duplications, and reported several inversion regions as palindromes (Regions #2 and #3). Chunking into 100 bp, 1 kbp or 10 kbp reads greatly improves fidelity of the alignments.

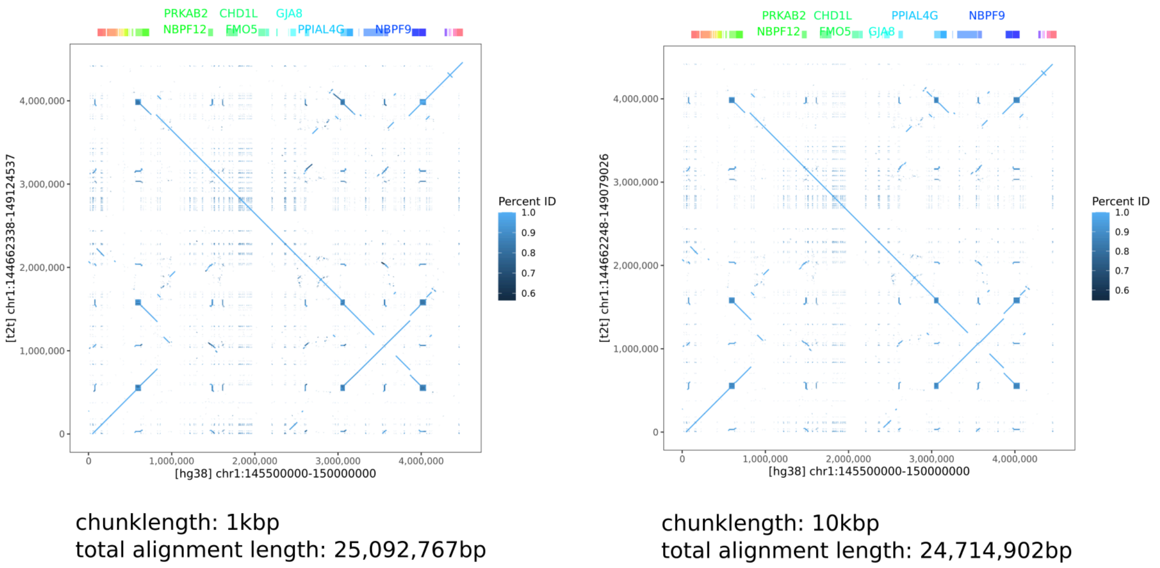

Fig S37. minimap2 alignments of the hg38 vs t2t references in one of the longest and most complex sSV regions located adjacent to the telomere of chr1. The chunklength parameter choice does not visually impact the discovery of off-diagonal segmental duplications.

1. Li, H. Minimap2: pairwise alignment for nucleotide sequences. *Bioinformatics* **34**, 3094–3100 (2018).
2. Yang, X. *et al.* Characterization of large-scale genomic differences in the first complete human genome. *Genome Biol.* **24**, 157 (2023).
