# Supplementary material for: Impact and characterization of serial structural variations across humans and great apes
Source: Nat Commun. 2024 Sep 13;15:8007. doi: 10.1038/s41467-024-52027-9 (PMC11393467; doi:10.1038/s41467-024-52027-9)
Supplement: Supplementary file 3 — Description of Additional Supplementary Files [file 41467_2024_52027_MOESM3_ESM.pdf]

Description of Additional Supplementary Files

File Name: Supplementary Data 1

Description: A table with coordinates and genotypes of 37 human sSVs across 56 haplotypes.

File Name: Supplementary Data 2

Description: A table with NAHRwhals calls in 336 CNV-associated regions of interest.

File Name: Supplementary Data 3

Description: A table with Genomic elements overlapping with sSVs (inversions, cups, mcnvs).

File Name: Supplementary Data 4

Description: A table with NAHRwhals calls in 48 morbid CNV-associated regions.
