# Supplementary material for: Impact and characterization of serial structural variations across humans and great apes
Source: Nat Commun. 2024 Sep 13;15:8007. doi: 10.1038/s41467-024-52027-9 (PMC11393467; doi:10.1038/s41467-024-52027-9)
Supplement: Supplementary file 5 — Reporting Summary [file 41467_2024_52027_MOESM5_ESM.pdf]

nature portfolio

Corresponding author(s): Prof. Jan O. Korbel

Last updated by author(s): 8th August 2024

Reporting Summary

Nature Portfolio wishes to improve the reproducibility of the work that we publish. This form provides structure for consistency and transparency in reporting. For further information on Nature Portfolio policies, see our Editorial Policies and the Editorial Policy Checklist.

Statistics

For all statistical analyses, confirm that the following items are present in the figure legend, table legend, main text, or Methods section.

n/a	Confirmed	
☐	☒	The exact sample size (*n*) for each experimental group/condition, given as a discrete number and unit of measurement
☐	☒	A statement on whether measurements were taken from distinct samples or whether the same sample was measured repeatedly
☐	☒	The statistical test(s) used AND whether they are one- or two-sided<br>*Only common tests should be described solely by name; describe more complex techniques in the Methods section.*
☐	☒	A description of all covariates tested
☐	☒	A description of any assumptions or corrections, such as tests of normality and adjustment for multiple comparisons
☐	☒	A full description of the statistical parameters including central tendency (e.g. means) or other basic estimates (e.g. regression coefficient) AND variation (e.g. standard deviation) or associated estimates of uncertainty (e.g. confidence intervals)
☐	☒	For null hypothesis testing, the test statistic (e.g. *F*, *t*, *r*) with confidence intervals, effect sizes, degrees of freedom and *P* value noted<br>*Give P values as exact values whenever suitable.*
☒	☐	For Bayesian analysis, information on the choice of priors and Markov chain Monte Carlo settings
☒	☐	For hierarchical and complex designs, identification of the appropriate level for tests and full reporting of outcomes
☒	☐	Estimates of effect sizes (e.g. Cohen's *d*, Pearson's *r*), indicating how they were calculated

Our web collection on statistics for biologists contains articles on many of the points above.

Software and code

Policy information about availability of computer code

Data collection	No new data has been collected for this study. All sequencing data (HiFi/Oxford Nanopore/Strand-Seq reads) used was produced as part of the Human Genome Structural Variation Consortium and can be accessed freely through their data portal. https://www.internationalgenome.org/data-portal/data-collection/structural-variation
Data analysis	We have exclusively used open-source, widely known bioinformatics software: minimap2 v2.18 , hifiasm v0.16, sniffles v2.3.3, samtools v 1.18, whatshap v2.4.<br>Software newly developed for this manuscript is also freely available (https://github.com/WHops/NAHRwhals) and depends only on published open-source software

For manuscripts utilizing custom algorithms or software that are central to the research but not yet described in published literature, software must be made available to editors and reviewers. We strongly encourage code deposition in a community repository (e.g. GitHub). See the Nature Portfolio guidelines for submitting code & software for further information.

Data

Policy information about availability of data

All manuscripts must include a data availability statement. This statement should provide the following information, where applicable:

- Accession codes, unique identifiers, or web links for publicly available datasets
- A description of any restrictions on data availability
- For clinical datasets or third party data, please ensure that the statement adheres to our policy

PacBio HiFi sequencing data, Strand-seq as well as Oxford Nanopore sequencing data were generated by the HGSVC consortium and can be accessed through the HGSVC data portal https://www.internationalgenome.org/data-portal/data-collection/structural-variation. Assembled genomes can be accessed via https://doi.org/10.5281/zenodo.7635935.

Research involving human participants, their data, or biological material

Policy information about studies with human participants or human data. See also policy information about sex, gender (identity/presentation), and sexual orientation and race, ethnicity and racism.

Reporting on sex and gender	All 28 included samples can -in principle- be assigned unambiguously to one biological sex based on gonosomes, with roughly equal numbers in the two groups (16 XX and 12 XY). In practice, biological sex has not been used as a discriminatory or phenotypic factor at any point of the analysis. Our findings are not specific to any sex. Gender, likewise, played no role in the analysis and has not been considered or even determined.
Reporting on race, ethnicity, or other socially relevant groupings	We used 'self-reported population supergroup' (AFR, AMR, EAS, EUR, SAS) labels based on the 1000 Genomes Project for a coarse initial ancestry grouping of participants in a small subset of analyses (e.g., Fig 2d, f). These categories were provided by participants, refer to broad geographic ancestry groups and have been surveyed by the 1000 Genomes Project during their initial data collection (https://www.internationalgenome.org/category/population). Each of the five 'superpopulation' labels is in turn an umbrella term for 4-6 sub-population groups which are listed under https://ftp.1000genomes.ebi.ac.uk/vol1/ftp/README_populations.md.<br><br>Our in-depth population-based analyses were always performed on actual SNP data and irrespective of population labels (Figures S26-S29). Our analysis did not consider social factors, and population labels were not used as proxies of any sort.
Population characteristics	The data used consitute a subsample of the 1000 Genomes Project, maximised for genomic diversity and balancing biological sex. Other factors such as age were not considered.
Recruitment	No participants were recruited by us. Recruitment of participants and data collection has been performed in the framework of the 1000 Genomes Project and its successor, the Human Genome Structural Variation Consortium.
Ethics oversight	See above, no new participants have been recruited

Note that full information on the approval of the study protocol must also be provided in the manuscript.

Field-specific reporting

Please select the one below that is the best fit for your research. If you are not sure, read the appropriate sections before making your selection.

☒ Life sciences ☐ Behavioural & social sciences ☐ Ecological, evolutionary & environmental sciences

For a reference copy of the document with all sections, see nature.com/documents/nr-reporting-summary-flat.pdf

Life sciences study design

All studies must disclose on these points even when the disclosure is negative.

Sample size	We have included 28 human samples
Data exclusions	No data has been excluded
Replication	N/A
Randomization	N/A
Blinding	N/A

Reporting for specific materials, systems and methods

We require information from authors about some types of materials, experimental systems and methods used in many studies. Here, indicate whether each material, system or method listed is relevant to your study. If you are not sure if a list item applies to your research, read the appropriate section before selecting a response.

Materials & experimental systems

n/a	Involved in the study
☒	☐ Antibodies
☒	☐ Eukaryotic cell lines
☒	☐ Palaeontology and archaeology
☒	☐ Animals and other organisms
☒	☐ Clinical data
☒	☐ Dual use research of concern
☒	☐ Plants

Methods

n/a	Involved in the study
☒	☐ ChIP-seq
☒	☐ Flow cytometry
☒	☐ MRI-based neuroimaging

Plants

Seed stocks	N/A
Novel plant genotypes	N/A
Authentication	N/A
